# Strawberry Notch 1 Acts as a Transcriptional Regulator Driving Oncogenic Programs in Liver Carcinogenesis

**DOI:** 10.1002/advs.202507238

**Published:** 2026-02-06

**Authors:** Sarah Fritzsche, Raisatun Nisa Sugiyanto, Kira Gür, Alina Krumme, Maxime Le Marois, Angelika Fraas, Aslihan Inal, Mario Huerta, Vanessa Henriques, Eva Eiteneuer, Thomas Albrecht, Alphonse Charbel, Michael T. Dill, Carsten Sticht, Carolina De La Torre, Stefan Pusch, Arianeb Mehrabi, Kai Breuhahn, Junfang Ji, Peter Schirmacher, Benjamin Goeppert, Stephanie Roessler

**Affiliations:** ^1^ Institute of Pathology, University Hospital Heidelberg Medical Faculty Heidelberg University Heidelberg Germany; ^2^ Laboratory of Macromolecular Engineering Department of Pharmaceutical Chemistry Faculty of Pharmacy Universitas Gadjah Mada Yogyakarta Indonesia; ^3^ Department of Gastroenterology Hepatology Infectious Diseases and Intoxications Heidelberg University Hospital Heidelberg Germany; ^4^ German Cancer Research Center (DKFZ) Heidelberg Experimental Hepatology Inflammation and Cancer Heidelberg Germany; ^5^ Liver Cancer Center Heidelberg (LCCH) Heidelberg Germany; ^6^ NGS Core Facility Medical Faculty Mannheim Heidelberg University Mannheim Germany; ^7^ Department of Neuropathology University Hospital Heidelberg Heidelberg Germany; ^8^ Clinical Cooperation Unit Neuropathology German Cancer Research Center Heidelberg Germany; ^9^ Department of General Visceral and Transplantation Surgery University Hospital Heidelberg Heidelberg Germany; ^10^ The MOE Key Laboratory of Biosystems Homeostasis & Protection Zhejiang Provincial Key Laboratory for Cancer Molecular Cell Biology and Innovation Center for Cell Signaling Network Life Sciences Institute Zhejiang University Hangzhou China; ^11^ Institute of Pathology Hospital RKH Kliniken Ludwigsburg Ludwigsburg Germany; ^12^ Institute of Tissue Medicine and Pathology University of Bern Bern Switzerland; ^13^ German Cancer Consortium (DKTK), DKFZ Core Center Heidelberg Germany

**Keywords:** cancer, cholangiocarcinoma, hepatocellular carcinoma, notch signaling, SBNO1

## Abstract

Aberrant Notch signaling has been identified as a key driver of cancer development. Genetic studies in *Drosophila* showed that the knockout of strawberry notch (*sno*) mimics the loss of *notch*. Here, we found that Strawberry Notch 1 (SBNO1) is upregulated in several cancer entities and elucidated the role of SBNO1 in liver cancer development. In hepatocellular carcinoma (HCC) and cholangiocarcinoma (CCA), SBNO1 protein was significantly increased and localized to the nucleus suggesting its involvement in gene regulation. SBNO1‐inhibition reduced cell viability, colony formation and migration and induced distinct expression patterns in HCC and CCA cell lines. However, BioID revealed that SBNO1 similarly modulates gene regulation in HCC and CCA by binding to general transcription factors TAF4 and TAF3. Deletion of *Sbno1* in murine liver cancer cells Hep55.1C reduced tumor growth in vivo. In addition, inhibition of Sbno1 significantly reduced liver tumor development in three different mouse models of HCC and CCA. Furthermore, *Sbno1*‐deletion reduced biliary differentiation and angiogenesis in the tumor margin, underscoring the necessity of Sbno1 in Notch‐driven CCA formation. Thus, we identified SBNO1 as a transcriptional regulator required for liver cancer development and progression.

## Graphical abstract

This study reports that SBNO1 protein is upregulated in several cancer entities. SBNO1 protein interacts with the basal transcription factor TFIID via TAF4, enabling its recruitment to transcription start sites and the modulation of target gene expression. In vivo and in vitro, SBNO1 protein is essential for liver cancer cell growth, highlighting its potential as a therapeutic target.

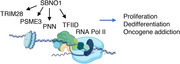

## 1 Introduction

Notch signaling has been demonstrated to be involved in the development of several cancer types [1]. In both liver cancer entities, hepatocellular carcinoma (HCC) and cholangiocarcinoma (CCA), Notch signaling is mainly tumor promoting but tumor suppressive effects have been observed in HCC [2, 3, 4, 5]. Liver cancer is a highly aggressive malignancy that poses a global health burden [6]. Accounting for about 80% of all liver cancer cases, HCC is the most common form of liver cancer, whereas CCA accounts for 10%–20% of cases [7, 8]. Based on the anatomical localization, CCA is classified into intrahepatic (iCCA), perihilar (pCCA) and distal CCA (dCCA). Recently, iCCA has been further stratified into small‐duct and large‐duct type iCCA, whereby small‐duct type iCCA is characterized by specific molecular alterations [9]. However, both HCC and CCA are very heterogenous diseases with regard to the clinicopathological presentation, therapy response, and molecular alterations [8, 10, 11]. Despite major advances in the field of genomic characterization of liver tumors, the prognosis for patients with liver cancer remains poor [6, 12]. Resection is still the only curative treatment but most patients present with advanced unresectable disease or insufficient liver function [13, 14]. Thus, a better understanding of the biology of HCC and CCA is required to develop effective therapies for advanced liver cancer.

Recently, we identified NOTCH pathway mutations and functionally characterized the direct NOTCH target gene *HES5* which acts as a transcriptional repressor in HCC [15]. Therapeutically, Crenigacestat (LY3039478) demonstrated its inhibitory role in Notch‐dependent angiogenesis in preclinical mouse models, and inhibition of fucosylation decreased Notch1 signaling by reduced binding of the NOTCH1 ligand Jagged1 [16, 17]. Mechanistically, NOTCH1 is cleaved upon ligand binding and the resulting NOTCH1 intracellular domain (N1ICD) translocates to the nucleus, where it acts as a transcriptional activator [18]. Interestingly, genetic studies in *Drosophila* showed that the knockout of strawberry notch (*sno*) mimics the *notch* knockout phenotype suggesting that sno is a downstream component of the Notch signaling pathway [19, 20]. However, the role of the human Strawberry Notch 1 (SBNO1) is poorly understood.

In this study, we found SBNO1 protein to be upregulated in several cancer entities including HCC and iCCA. SBNO1‐inhibition reduced cell viability, colony formation and migration in vitro. Furthermore, we demonstrated in four different liver cancer mouse models that Sbno1 is required for HCC and iCCA development. Mechanistically, SBNO1‐knockdown induced distinct expression patterns in HCC and CCA cell lines but SBNO1 similarly modulates gene regulation in HCC and CCA by binding to general transcription factors. Taken together, SBNO1 is essential for HCC and CCA cell growth and may be exploited therapeutically.

## 2 Results

### 2.1 SBNO1 is Overexpressed in Liver Cancer

**FIGURE 1:**
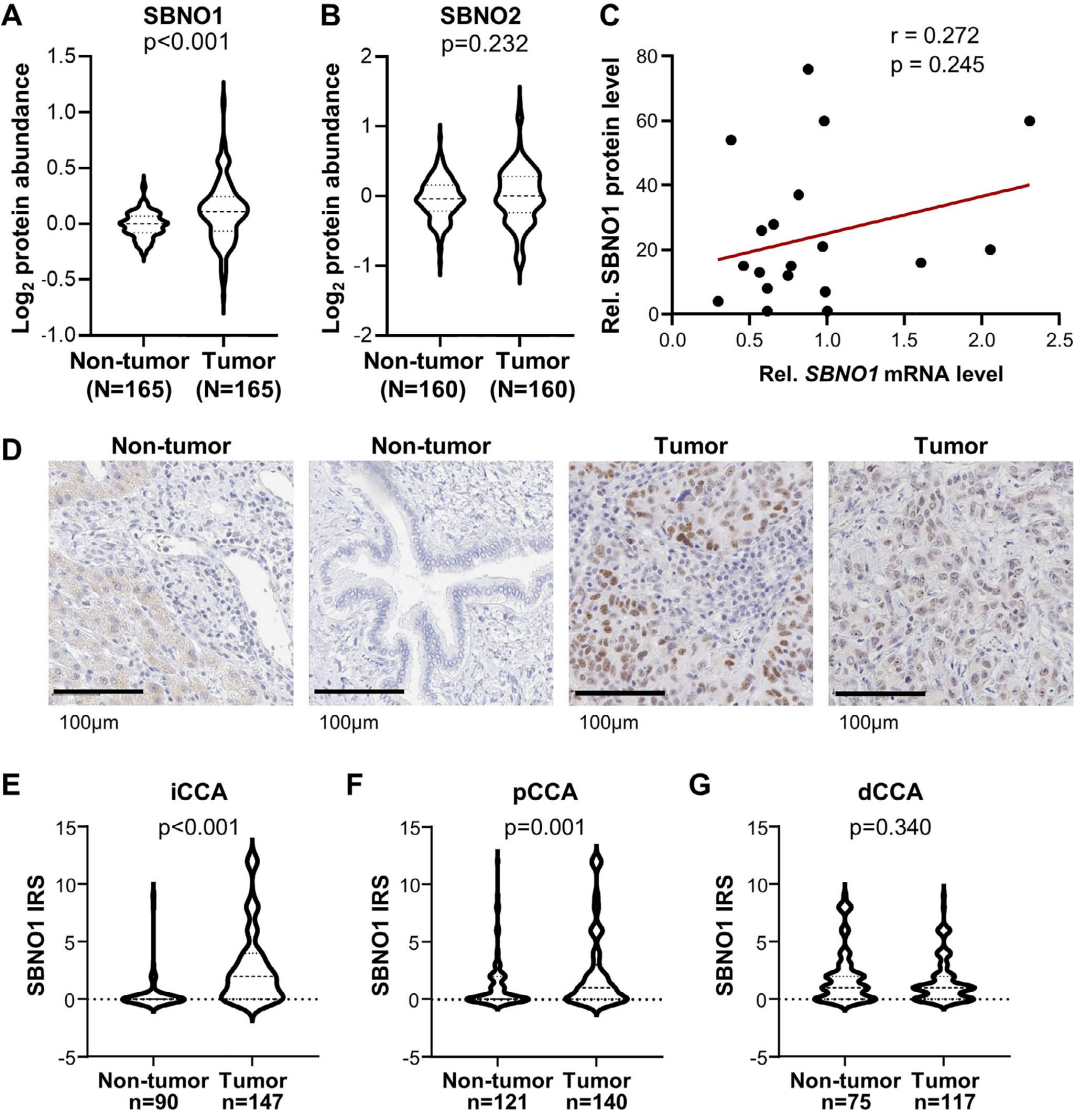
SBNO1 protein but not mRNA is upregulated in liver cancer patients. (A) SBNO1 and (B) SBNO2 protein expression in the tumor and paired adjacent non‐tumor tissues of 165 HBV‐HCC patients is depicted (Proteomic Data Commons PDC000198; https://proteomic.datacommons.cancer.gov/pdc/) Median centered log_2_ protein abundance of tumor and non‐tumor samples is shown. SBNO2 protein was undetectable in five patients. (C) SBNO1 protein and mRNA levels do not correlate in human liver cancer cell lines (*n* = 18) and the immortalized hepatocyte and cholangiocyte cell lines, HHT4 and NHC. (D) Representative images of two liver samples without neoplastic alterations (non‐tumor) and two different iCCA samples (tumor) immunohistochemically stained with an anti‐SBNO1 antibody. (E) The SBNO1 expression is shown as violin plots of the respective IRS scores in non‐tumor and tumor samples of patients with iCCA, (F) pCCA or (G) dCCA. *P*‐values were calculated using unpaired Student's t‐test.

To study the role of Strawberry Notch 1 (SBNO1) and 2 (SBNO2) in carcinogenesis, we analyzed the publicly available TCGA datasets and found that SBNO1 is overexpressed in several cancer entities (Figure S1). In gastrointestinal cancers including esophageal carcinoma (ESCA), stomach adenocarcinoma (STAD) and cholangiocarcinoma (CHOL), and in diffuse large B‐cell lymphoma (DLBC) and thymoma (THYM) *SBNO1* mRNA was higher in the tumor compared to non‐tumor samples (Figure S1A,B). In contrast, *SBNO2* mRNA was elevated in CHOL and pancreatic adenocarcinoma (PAAD) but reduced in lung squamous cell carcinoma (LUSC), DLBC and THYM (Figure S1C,D). In a large dataset of patients with iCCA, *SBNO2* but not *SBNO1* mRNA levels were elevated in iCCA compared to non‐tumor tissue samples (GSE26566; Figure S2A,B) [21]. However, *SBNO1* was overexpressed and *SBNO2* was repressed in eCCA compared to non‐tumor tissue (GSE132305; Figure S2C,D) [22]. Therefore, we next evaluated the protein expression and found that SBNO1 but not SBNO2 protein was upregulated in proteomics data of paired HCC tumor compared to non‐tumor tissue samples (PDC000198; Figure 1A,B). Furthermore, high SBNO1 protein levels were associated with significantly shorter overall survival and a trend of shorter disease‐free survival which was mainly associated with protein but not mRNA levels (Figure S2E–G). Correlation of *SBNO1* mRNA and protein levels in 18 liver cancer cell lines and the immortalized hepatocyte and cholangiocyte cell lines, HHT4 and NHC, revealed that mRNA and protein levels only partially correlated (Figure 1C; Figure S3A,B). Similarly, no clear correlation was observed in the subsets of HCC or CCA cells, respectively (Figure S3C,D). As *SBNO2* mRNA and protein levels did not show increased expression in HCC, we analyzed SBNO1 protein expression in a tissue microarray including non‐neoplastic normal bile duct and CCA. Interestingly, immunohistochemical staining of SBNO1 protein revealed that SBNO1 localized to the nucleus of tumor epithelial cells suggesting a role in gene regulation (Figure 1D). SBNO1 protein was increased in iCCA and pCCA but not dCCA compared to the corresponding non‐neoplastic bile duct epithelium (Figure 1E–G). Thus, SBNO1 was significantly increased at the mRNA and/or protein level in several cancer entities suggesting an oncogenic function in liver carcinogenesis.

### 2.2 SBNO1 Protein Repression Reduces Liver Cancer Cell Viability, Clonogenicity and Migration

**FIGURE 2:**
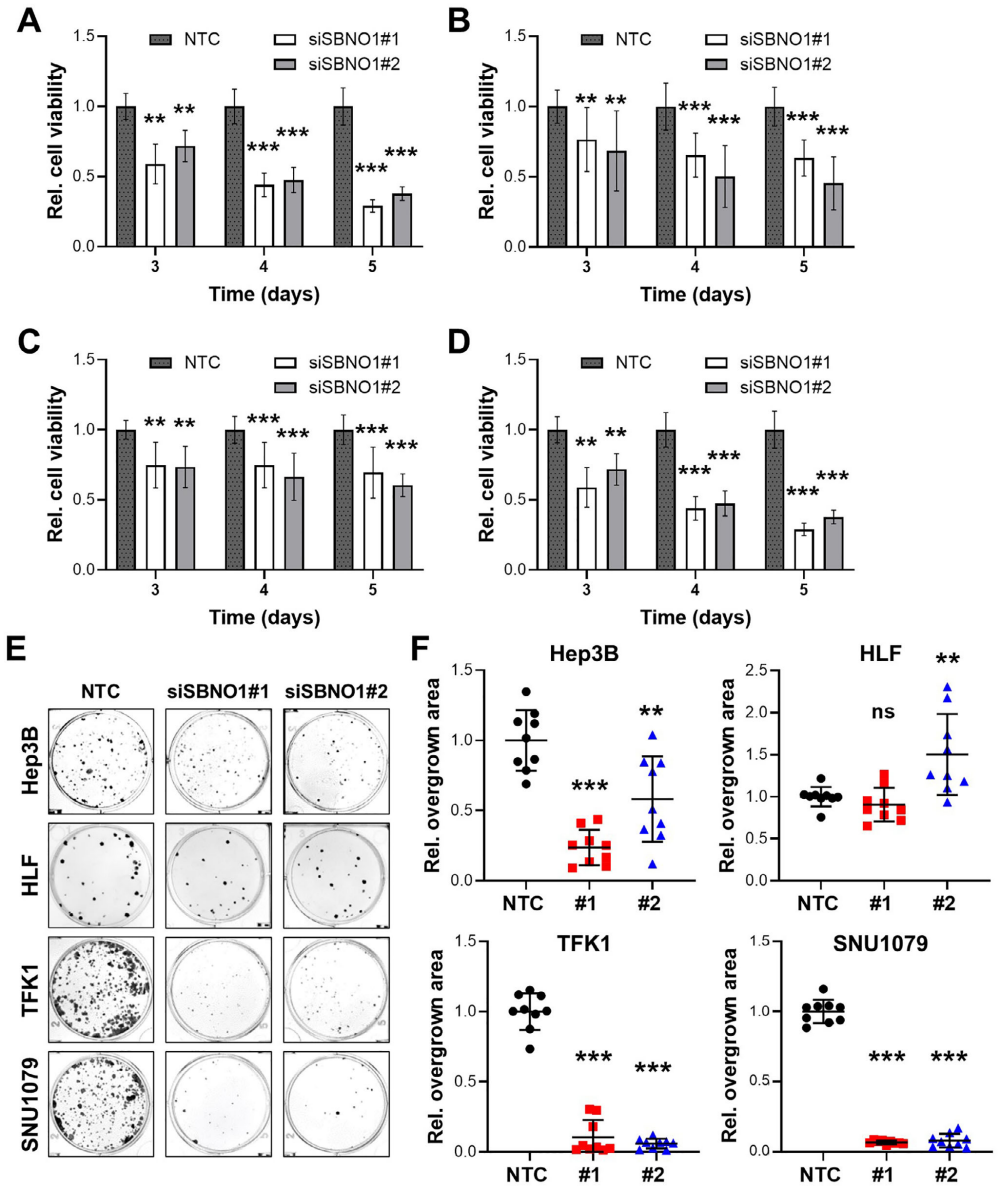
Inhibition of SBNO1 reduces cell viability and clonogenicity in HCC and CCA cell lines. (A) Cell viability upon knockdown of SBNO1 in Hep3B (*n* = 3), (B) HLF, (C) TFK1 (*n* = 3) and (D) SNU1079 (*n* = 3). (E) Representative images and (F) quantitative analysis of colony formation assays of Hep3B, HLF, TFK1 and SNU1079 upon siRNA‐mediated SBNO1 knockdown (*n* = 3). *P*‐values were calculated using unpaired Student's t‐test. ns: not significant; ^**^ *p* < 0.01; ^***^ *p* < 0.001.

To decipher the function of SBNO1 in liver carcinogenesis, we selected the human HCC cell lines HLF and Hep3B, as well as the human CCA cell lines SNU1079 and TFK‐1, as they endogenously express SBNO1 protein (Figure S3A,B). To repress SBNO1 protein levels, two different siRNA were tested for efficient knockdown and used in functional assays (Figure S3E,F). Inhibition of SBNO1 expression resulted in significantly reduced cell viability in all four cell lines (Figure 2A–D). Furthermore, colony formation assays demonstrated a significant reduction of clonogenicity upon SBNO1 protein knockdown in Hep3B, TFK‐1 and SNU1079 cells (Figure 2E,F).

**FIGURE 3:**
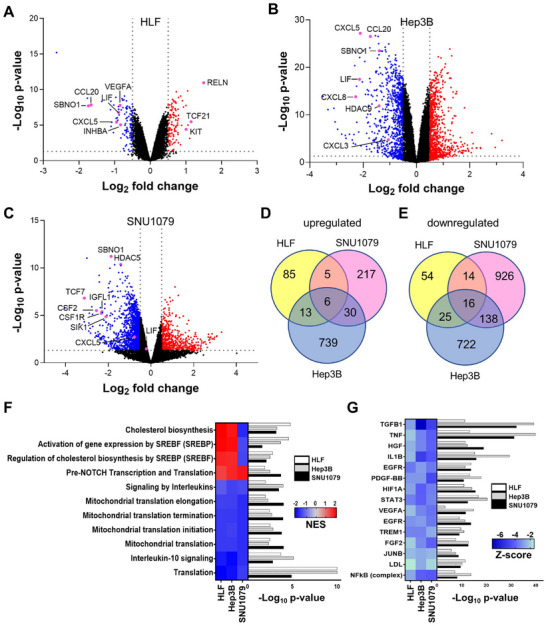
Inhibition of SBNO1 leads to inhibition of oncogenic programs. (A) Volcano plot showing the gene expression differences in HLF, (B) Hep3B and (C) SNU1079 cells upon inhibition of SBNO1 by siRNA compared to control siRNA (NTC). Significantly (*P* < 0.05) downregulated genes with log_2_FC (log_2_‐fold change) < ‐0.5 are indicated in blue and upregulated genes log_2_FC > 0.5 are depicted in red. (D) Overlap of significantly upregulated (FC > 0.5, adjusted *P* < 0.05) and (E) downregulated (FC < ‐0.5, adjusted *P* < 0.05) genes in HLF, Hep3B and SNU1079 cells. (F) Reactome pathways significantly altered by inhibition of SBNO1 in HLF, Hep3B, and SNU1079 cells upon SBNO1 knockdown. Normalized Enrichment Scores (NES) are indicated by color coding. (G) Upstream regulators inferred by the gene expression changes in HLF, Hep3B, and SNU1079 cells upon SBNO1 knockdown were analyzed by Ingenuity Pathway Analysis. Z‐scores are indicated by color coding.

To dissect the pathways altered by SBNO1 knockdown, we performed gene expression analysis of HLF, Hep3B and SNU1079 cells upon siRNA‐mediated SBNO1 knockdown. Differential gene expression analysis revealed many genes to be dysregulated and the most‐strongly downregulated genes included cytokines in all three cell lines (Figure 3A–C). As only few genes were similarly upregulated or downregulated (Figure 3D,E), we performed qRT‐PCR analysis for independent validation of the gene expression profiling. First, we analyzed *LIF* which was among the downregulated genes in HLF and Hep3B, whereas it was unaltered in SNU1079 (Figure 3A–C). Consistently, the same expression alteration patterns were detected by qRT‐PCR (Figure S4A,B). Furthermore, we analyzed the mRNA expression of *RELN*, *TCF21*, and *KIT* which were among the most upregulated genes in HLF cells only (Figure 3A–C). Similarly, qRT‐PCR confirmed the significant upregulation of these three genes in HLF cells upon siRNA‐mediated knockdown of SBNO1 (Figure S4C). Next, we performed downstream pathway analysis and found that in all three cell lines, “pre‐NOTCH Transcription and Translation” was enriched, whereas cholesterol biosynthesis and gene expression by SREBP/SREBF were only enriched in the two HCC cell lines HLF and Hep3B (Figure 3F). In addition, interleukin signaling and mitochondrial translation were significantly repressed in all three cell lines (Figure 3F). Next, we sought to study the potential upstream regulators of the observed gene expression profiles. Therefore, we inferred the upstream regulators using the bioinformatic tool Ingenuity Pathway Analysis. Consistent with the observed reduction of interleukin signaling, upstream regulators involved in inflammation and interleukin signaling, such as TGFB1, IL1B, EGFR, STAT3, and the NFκB complex, were reduced in all three cell lines upon SBNO1 protein knockdown (Figure 3G). The inhibition of SBNO1 protein revealed that SBNO1 protein knockdown reduced cell viability and clonogenicity of HCC and CCA cells and repressed interleukin and mitochondrial signaling.

### 2.3 Inhibition of Sbno1 Reduces Tumor Cell Growth In Vitro and In Vivo

**FIGURE 4:**
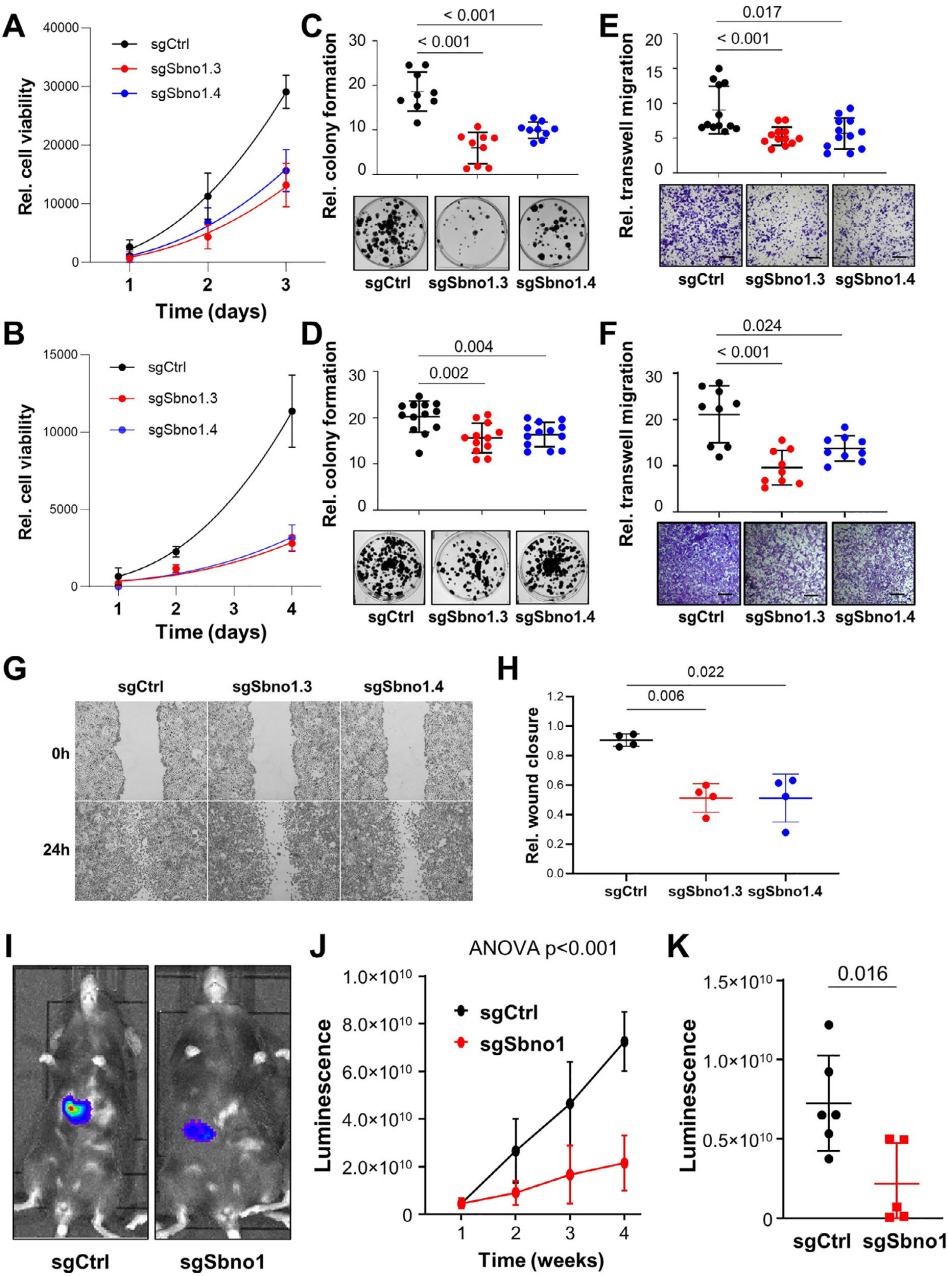
CRISPR/Cas9 knockout of *Sbno1* reduces tumor cell growth and migration in vitro and in vivo. (A) Cell viability upon *Sbno1* knockout by sgSbno1.3 or sgSbno1.4 in Hep56.1D and (B) Hep55.1C cells. (C) Quantitative analysis (top) and representative images (bottom) of colony formation of Hep56.1D and (D) Hep55.1C cells are shown. (E) Quantitative analysis (top) and representative images (bottom) of transwell migration assays of Hep56.1D and (F) Hep55.1C cells are depicted. (G) Scratch migration assay of Hep56.1D cells showing representative images and (H) quantitative analysis. (I) IVIS imaging results after intrahepatic tumor injection of Hep55.1C‐Luc:GFP cells transduced with control sgRNA (sgCtrl) or sgSbno1.3. (J) Cumulative tumor growth was monitored by bioluminescence imaging (BLI) and reported in photons/s/cm^2^/steradian (p/s/cm^2^/sr). (K) Luminescence of tumors at 4 weeks after injection was monitored by BLI. Mean values with SD and unpaired Student's t‐test *P‐*values are depicted.

Next, we analyzed the effect of *Sbno1* repression by CRISPR/Cas9‐mediated knockout in the murine cell lines Hep56.1D and Hep55.1C. Both cell lines were derived from N‐nitrosodiethylamine‐induced liver tumors of C57BL/6J mice enabling their study in immune competent C57BL/6J mice [23]. To obtain efficient *Sbno1* knockout, we tested 4 different sgRNA and observed by Western blot the highest knockout with significant reduction of Sbno1 protein using sgSbno1.3 and sgSbno1.4 (Figure S5). Similar to inhibition of SBNO1 in human cell lines, knockout of *Sbno1* using two different sgRNA reduced cell viability in Hep56.1D and Hep55.1C (Figure S5, Figure 4A,B). Furthermore, we studied the effect of Sbno1 on cell clonogenicity. Colony formation was also significantly reduced in both cell lines (Figure 4C,D). To test if Sbno1 is involved in cell migration, we performed transwell migration and scratch migration assays. In transwell migration assays, Hep56.1D and Hep55.1C cells both migrated significantly less after sgRNA‐mediated knockout of *Sbno1* (Figure 4E,F). In scratch migration assays, Hep55.1C control cells did not form a migration front into the scratch and thus, only Hep56.1D cells were studied. Hep56.1D cells transduced with control sgRNA closed the scratch almost completely within 24 h, whereas cells with *Sbno1* knockout (sgSbno1.3 and sgSbno1.4) showed a significantly reduced closure of the scratch (Figure 4G,H). Thus, repression of Sbno1 protein led to significantly reduced cell viability, clonogenicity and migration of murine liver cancer cells in vitro.

As *Sbno1* knockout significantly reduced liver cancer cell viability and colony formation, Hep55.1C cells expressing luc:GFP‐fusion protein were injected into the livers of syngeneic C57BL/6J wildtype mice and tumor growth was monitored in vivo using bioluminescence imaging (BLI). Tumors of Hep55.1C cells transduced with sgSbno1 exhibited significantly reduced tumor growth over time compared to control cells transduced with sgCtrl (Figure 4I,J). At the end point after four weeks the luminescence signal was significantly reduced in mice injected with sgSbno1 cells compared to sgCtrl (Figure 4K). Thus, SBNO1 protein is required for liver cancer cell viability, clonogenicity, and cell migration in vitro and in vivo.

### 2.4 SBNO1 is Required for HCC and CCA Tumor Initiation In Vivo

**FIGURE 5:**
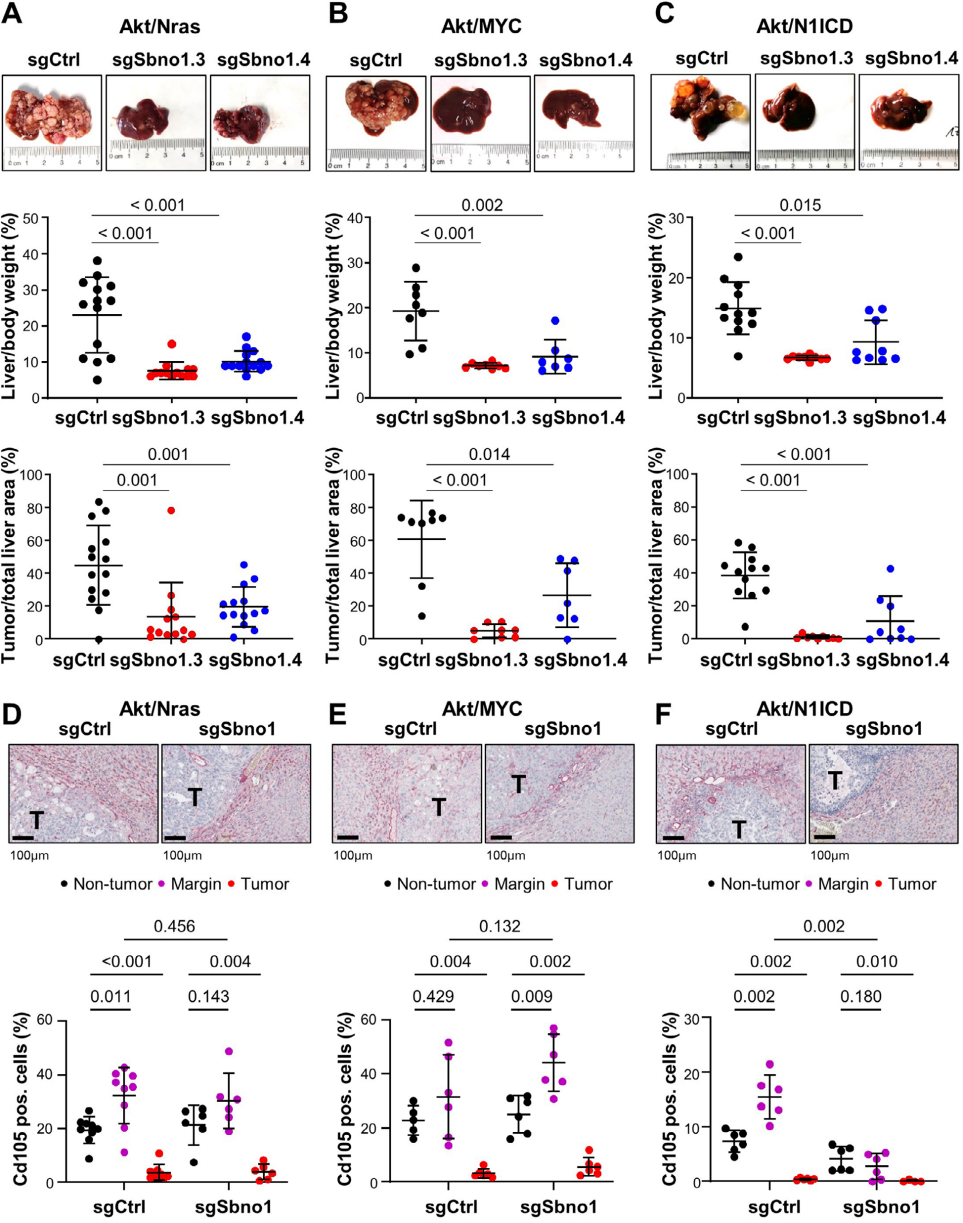
Sbno1 is required for HCC and CCA tumor formation in three independent mouse models. (A–C) Representative images of murine livers (top panel), liver‐to‐body weight ratio (middle panel) and tumor‐to‐total liver area (bottom panel) are shown. (A) Mice were transduced with myrAkt/Nras‐G12V (Akt/Nras) together with sgCtrl (*n* = 14), sgSbno1.3 (*n* = 13) or sgSbno1.4 (*n* = 14). Livers were collected 9–11 weeks after injection. (B) Murine livers transduced with Akt/MYC together with sgCtrl (*n* = 8), sgSbno1.3 (*n* = 8) or sgSbno1.4 (*n* = 7). Livers were collected 5 weeks after injection. (C) Shown are livers transduced with Akt/N1ICD together with sgCtrl (*n* = 12), sgSbno1.3 (n = 9) or sgSbno1.4 (*n* = 9). Livers were collected 8 weeks after injection. (D) Representative images of anti‐Cd105 staining of Akt/Nras‐G12V, (E) Akt/MYC and (F) Akt/N1ICD transduced livers together with sgCtrl or sgSbno1 (upper panel). T, tumor. The percentages of Cd105‐positive cells are plotted as scatter plot in the non‐tumor, tumor margin and tumor areas (bottom panel). sgSbno1.3 and sgSbno1.4 were combined into one group. Mann–Whitney U‐test *P*‐values are depicted.

For validation of the findings observed in human and murine cell lines and to further study liver cancer development in vivo, we applied three different orthotopic mouse models using hydrodynamic tail vein injection (HDTV). The first model used Akt and Nras‐G12V to induce HCC [24, 25], in the second mouse model, expression of Akt/MYC results in low‐grade HCC [26] and last, Akt/N1ICD leads to iCCA formation [4]. In all three liver cancer models, we asked whether sgSbno1‐mediated knockout of *Sbno1* would affect liver tumorigenesis. We found that livers of mice with knockout of *Sbno1* using two different sgRNA showed significantly reduced tumor development compared to the control Cas9/sgRNA vector (sgCtrl; Figure 5A–C). The liver‐to‐body weight ratios of mice transduced with Akt/Nras/sgCtrl, Akt/MYC/sgCtrl or Akt/N1ICD/sgCtrl were significantly higher compared to the respective liver‐to‐body weight ratios of mice transduced with sgSbno1.3 or sgSbno1.4 (Figure 5A–C). In addition, histological analysis of mouse livers was performed. The tumor‐to‐total liver area was significantly reduced upon transduction with sgSbno1.3 or sgSbno1.4 in all three liver cancer mouse models (Figure 5A–C). The number of tumors per mouse was partially reduced by *Sbno1* knockdown suggesting reduced tumor growth and initiation (Figure S6A–C). To further characterize the tumors with or without sgSbno1, we stained for Ki67 and observed that the tumors of mice injected with sgSbno1 had similar proliferation compared to tumors of mice injected with sgCtrl suggesting that the small tumors growing in sgSbno1‐transduced tumors overcame *Sbno1* knockout (Figure S7A–C). In addition, the number of infiltrating Cd11b‐positive macrophages, key mediators of inflammation in liver carcinogenesis, was similar in tumors with sgSbno1 compared to sgCtrl in all three mouse models (Figure S7D–F). Thus, Sbno1 protein is required for HCC and iCCA tumor initiation in vivo.

As previously observed, the majority of Akt/Nras tumors were histologically classified as HCC but about 10% of tumors showed CCA or mixed HCC‐CCA histology [24]. Also, in the few tumors which did develop in mice injected with sgSbno1, a small proportion of tumor cells expressed Ck7 and Ck19 (Figure S8A,B). In contrast, the Akt/MYC mouse model resulted in dedifferentiated HCC with patchy Ck7 and Ck19 expression. Tumors of mice injected with sgSbno1 had reduced expression of Ck7 and Ck19 suggesting that Sbno1 is required for development of dedifferentiated HCC (Figure S8C,D) [27]. Similarly, Akt/N1ICD‐induced iCCA tumor development and these tumors expressed Ck7 and Ck19 (Figure S8E,F). Knockout of *Sbno1* resulted in significantly reduced expression of Ck7 and Ck19 compared to the control group (Figure S8E,F). Furthermore, we analyzed the expression of endoglin (Cd105) to study the neoangiogenesis in the three liver cancer mouse models. We observed Cd105‐positive endothelial cells in the tumor surrounding liver of all three mouse models. In addition, all control mice exhibited an increase of Cd105‐positive cells in the tumor margin and very low to absent Cd105 staining in the tumor (Figure 5D–F). No differences in Cd105‐positive cells of the non‐tumor, margin and tumor areas between sgSbno1 and sgCtrl were observed in the Akt/Nras and Akt/MYC mouse models (Figure 5D,E). But we found that the iCCA mouse model of Akt/N1ICD tumors exhibited a lower number of Cd105‐positive endothelial cells in the tumor margin upon sgSbno1 transduction compare to Akt/N1ICD tumors with sgCtrl (Figure 5F). Thus, Sbno1 is required for CCA tumor differentiation and inhibition of Sbno1 in CCA led to reduced neoangiogenesis at the tumor margin.

### 2.5 SBNO1 Inhibition Is Not Toxic to Normal Hepatocytes

**FIGURE 6:**
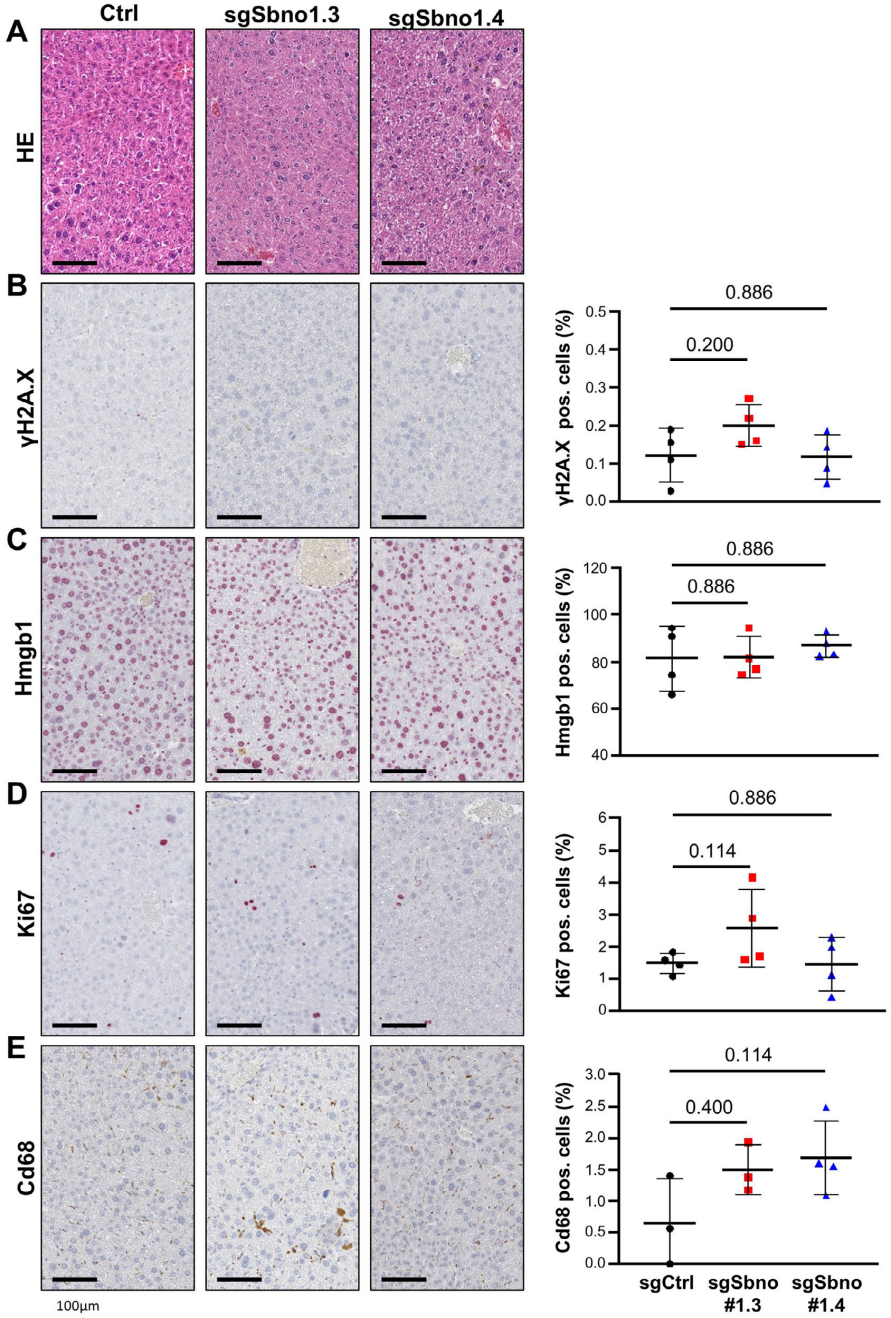
Deletion of Sbno1 protein in the liver does not induce cytotoxicity in vivo. (A) HE staining of mouse livers one week after *Sbno1* knockout by sgSbno1.3 or sgSbno1.4. (B) Representative images of immunohistochemical staining of H2A.X phosphorylated at Ser139 (γH2A.X), (C) Hmgb1, (D) Ki67 and (E) Cd68 (left panel) and quantification of positively stained cells represented as scatter plot (right panel). HE, hematoxylin and eosin. Mann–Whitney U‐test *P*‐values are depicted.

To explore the potential inhibition of SBNO1 in liver cancer patients, we investigated the knockout of *Sbno1* in hepatocytes as varying expression levels of SBNO1 were observed in murine and human hepatocytes (https://www.livercellatlas.org; GSE192742, Figure S9). The two independent sgRNA targeting *Sbno1*, sgSbno1.3 and sgSbno1.4, or sgCtrl were introduced into hepatocytes by hydrodynamic injection of mice and one week after transduction, livers were analyzed. No changes in histology using hematoxylin and eosin (HE) staining or in liver‐to‐body weight ratio were observed (Figure 6A; Figure S10). In addition, the apoptosis marker γ‐H2A.X and the necrosis‐associated protein Hmgb1 were not significantly altered (Figure 6B,C). As hepatocyte damage results in proliferation of hepatocytes and hepatic progenitor cells, we furthermore evaluated the expression of Ki67. The number of Ki67‐positive cells was not significantly different between mice injected with sgCtrl compared to sgSbno1.3 or sgSbno1.4 (Figure 6D). The number Cd68‐positive macrophages indicating inflammation was also comparable between sgCtrl and sgSbno1.3 or sgSbno1.4 (Figure 6E). Therefore, knockout of *Sbno1* in hepatocytes did not lead to apoptosis, necrosis or inflammation in mice.

### 2.6 SBNO1 Interacts with Transcription Factors and the Basal Transcription Factor TFIID

**FIGURE 7:**
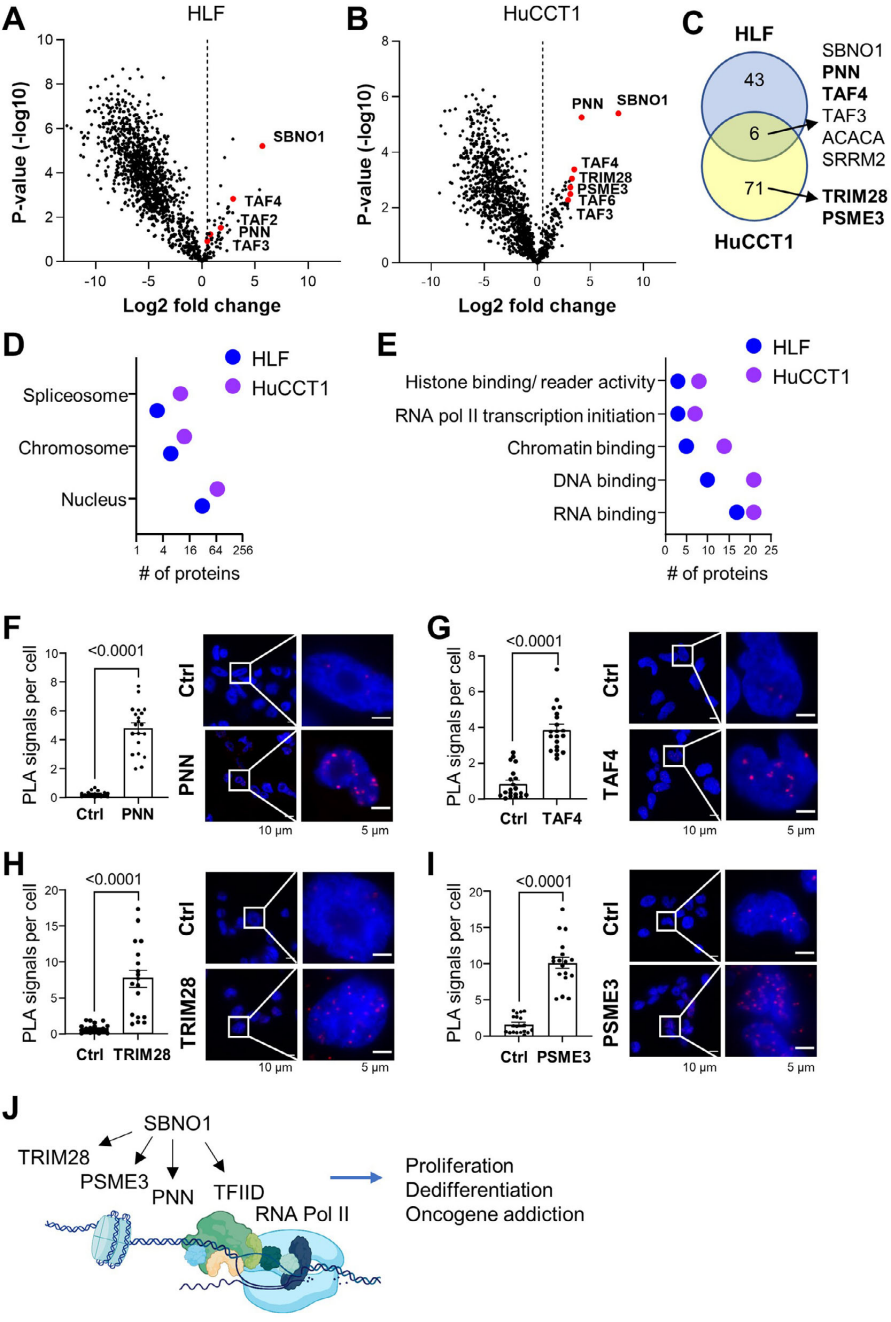
SBNO1 protein interacts with transcriptional regulators. (A) Volcano plot depicting enrichment of proteins biotinylated upon expression of SBNO1‐BirA fusion protein compared to BirA‐Ctrl in HLF and (B) HuCCT1 cells. (C) Intersection of significantly enriched proteins in HLF and HuCCT1 cells (log_2_FC≥0.5 and q‐value<0.05). Proteins highlighted in bold were selected for further analysis. (D) Significantly biotinylated proteins with log_2_FC>0.5 were analyzed using DAVID (https://davidbioinformatics.nih.gov/). Shown are the significantly enriched cellular components and (E) molecular functions. (F) Representative images of proximity ligation assays (PLA) for interaction of SBNO1 with PNN, (G) TAF4 and (H) TRIM28 and (I) PSME3 in HuCCT1 cells. Quantification of PLA dots per cell by scatter plots with mean and SD. *N*  =  20 cells of 8 images analyzed for each condition. Mann–Whitney U‐test *P*‐values are shown. (J) Schematic diagram showing the interaction of SBNO1 with transcriptional regulators and the basal transcription factor TFIID, created in BioRender. Roessler, S. (2025) https://BioRender.com/fm3X52s.

Next, we aimed to unravel the mechanism of SBNO1 protein function in liver cancer. We performed BioID which is a method of proximity labeling using the biotin ligase BirA to identify SBNO1 interaction partners [28]. BirA was fused to the C‐terminus of SBNO1 and BirA alone served as control. The inducibility and functional expression of the doxycycline (Dox)‐inducible fusion protein SBNO1‐BirA or control BirA (BirA‐Ctrl) were confirmed by Western blot upon treatment with or without Dox and biotin (Figure S11A). The addition of biotin led to biotinylation of proteins in the proximity of the SBNO1‐BirA and random biotinylation in BirA‐Ctrl (Figure S11B–D). HLF and HuCCT1 cells which express intermediate levels of SBNO1 protein were used for the BioID assay (Figure S3). Biotinylated proteins were pulled down by streptavidin and subjected to mass spectrometric quantification (Figure S11C,D). In HLF cells, 49 proteins were significantly more abundant in the SBNO1‐BirA group compared to the BirA‐Ctrl (log_2_FC > 0.5, q‐value ≤ 0.05; Figure 7A; Table S1) and in HuCCT1, 77 proteins were significantly enriched by SBNO1‐BirA compared to the control (log_2_FC > 0.5, q‐value ≤ 0.05; Figure 7B; Table S2). Surprisingly, only 6 proteins including SBNO1 itself were in common between the two cell lines suggesting cell‐type specific molecular mechanisms (Figure 7C). Among the common potential interaction partners of SBNO1 were TAF4 (TATA‐box binding protein‐associated factor 4) and TAF3 which are part of the general transcription factor TFIID, a component of the RNA polymerase II pre‐initiation complex that interacts with tissue‐specific transcription factors to regulate gene expression [29]. PNN (pinin) is involved in mRNA splicing and interacts with the transcriptional co‐repressor CTBP1 (c‐terminal binding proteins 1) regulating gene expression [30]. In addition, TRIM28 and PSME3 were among the SBNO1‐interaction partners detected in HuCCT1 but not HLF cells (Figure 7C). TRIM28 (also known as TIF1β) has multiple functions including transcriptional co‐factor regulating chromatin organization, E3 ubiquitin ligase targeting proteins for proteasomal degradation and E3 SUMO ligase mediating gene repression [31]. TRIM28 promoted cell proliferation by bridging HDAC1 thereby increasing E2F3 and E2F4 DNA binding [32]. PSME3 (also named PA28ϒ) is a proteasomal regulator which has been shown to promote MDM2‐mediated degradation of p53 [33] and to regulate cell cycle progression via the transcription factor CP2 (TFCP2) [34]. Further analysis of the SBNO1 interaction partners revealed that most proteins exhibited localization to the nucleus, chromosomes or spliceosomes and had a molecular function in RNA binding, DNA binding, chromatin binding, RNA polymerase II transcription initiation or histone binding/reader activity (Figure 7D,E; Figure S12). This suggested that SBNO1 may act as transcriptional regulator via interaction with TAF4, TAF3 and PNN and to modulate gene expression via regulation of cell‐type or cell‐function specific transcription factors.

For independent validation of SBNO1‐interaction partners, in situ proximity ligation assay (PLA) was performed for PNN, TAF4, TRIM28 and PSME. SBNO1 and all potential SBNO1‐interaction partners except for TAF4 exhibited exclusively nuclear localization (Figure S13). Consistent with previous reports, TAF4 localized to the nucleus and cytoplasm [35]. In the PLA, SBNO1‐complexes with PNN, TAF4, TRIM28, and PSME localized to the nucleus in iCCA cell lines HuCCT1 and SNU1079 (Figure 7F–I; Figure S14). Therefore, SBNO1 protein may function as a modulator of transcriptional regulation by interaction with the basal transcription factor TFIID and indirectly through additional factors including PNN, TRIM28 and PSME (Figure 7J).

### 2.7 SBNO1 and TAF4 Exhibit Similar Chromatin Binding Patterns

**FIGURE 8:**
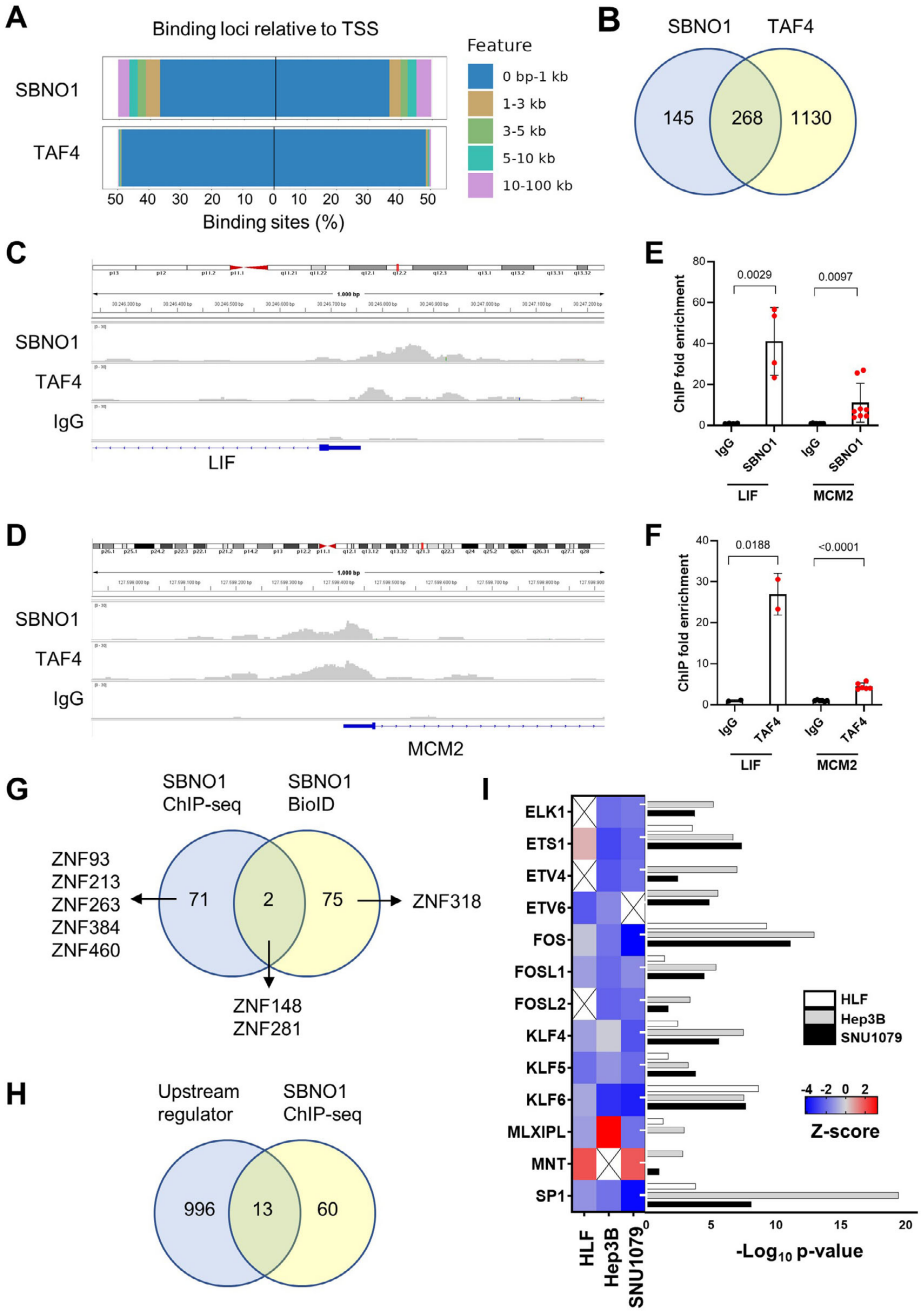
Chromatin immunoprecipitation sequencing (ChIP‐seq) of SBNO1 and TAF4 shows overlapping target gene binding at the transcriptional start site (TSS). (A) Binding loci distribution of ChIP‐seq peaks of SBNO1 and TAF4. (B) Intersection of significantly bound promoters (within 1 kb of the TSS) by SBNO1 and TAF4 ChIP‐seq. (C) ChIP‐Seq profile of the human *LIF* and (D) *MCM2* gene loci for SBNO1, TAF1 and the IgG control. (E) SBNO1 and (F) TAF4 ChIP‐qPCR detecting the genomic area of the TSS of *LIF* and *MCM2*. Mann–Whitney U‐test *P*‐values are depicted. (G) Intersection of potential SBNO1 interaction partners identified by BioID and of transcription factor binding motifs identified in the SBNO1 ChIP‐seq peaks. (H) Intersection of upstream regulators detected in at least two of three cell lines (HLF, Hep3B and SNU1079), and of transcription factor binding motifs identified in the SBNO1 ChIP‐seq peaks. (I) Enrichment of the 13 upstream regulators which have potential binding sites in SBNO1 ChIP‐seq peaks (intersection of H). Z‐scores were inferred by the gene expression changes in HLF, Hep3B and SNU1079 cells upon SBNO1 knockdown were analyzed by Ingenuity Pathway Analysis. Z‐scores are indicated by color coding.

To study the interaction of SBNO1 and TAF4, we performed chromatin immunoprecipitation followed by sequencing (ChIP‐seq) in HuCCT1 cells. Analysis of the peak count frequency along the genomic region of genes revealed globally an enrichment of ChIP‐seq peaks around the transcription start site (TSS) for SBNO1 and TAF4 (Figure S15A,B). As expected, 97% of TAF4 ChIP peaks were detected within 1 kb from the TSS (Figure 8A). SBNO1 also bound primarily to the genomic area close to the TSS with 73% of the SBNO1 ChIP peaks located within 1 kb from the TSS (Figure 8A). Next, we focused on the genes which were bound by SBNO1 or TAF4 in their promoter region (within 1 kb of the TSS). Of the gene promoters bound by SBNO1, 65% (268 of 413) of genes were observed to be also bound by TAF4 suggesting that SBNO1 mainly interacted with TAF4, besides 19% (268 of 1398) of the TAF4‐bound genes were also bound by SBNO1 (Figure 8B). As examples, we tested if *LIF* and *MCM2*, which have been shown to be involved in liver cancer cell proliferation and aggressive behavior [36, 37], might be regulated by SBNO1. Both *LIF* and *MCM2* were downregulated in HuCCT1 cells after SBNO1 knockdown (Figure S15C,D). The SBNO1 and TAF4 ChIP‐seq data showed enrichment around the TSS of *LIF* and *MCM2* and ChIP‐qPCR of primers spanning the TSS of *LIF* and *MCM2* demonstrated significant enrichment compared to the IgG control indicating that SBNO1 and TAF4 may directly regulate the expression of *LIF* and *MCM2* (Figure 8C–F). To better characterize the SBNO1 ChIP‐seq peaks, we analyze the genomic sequence of the chromatin bound by SBNO1. Interestingly, the SBNO1 ChIP‐seq peaks were enriched for binding sites of zinc finger proteins (ZNF) and two of them, ZNF148 and ZNF281, were also identified as potential SBNO1 interaction partners by BioID (Figure 8G). Furthermore, 13 of the transcription factors with binding sites in the SBNO1 ChIP‐seq peaks were also identified as upstream regulators in at least two of the cell lines analyzed by gene expression profiling (Figure 8H). These 13 transcription factors included several ETS, FOS, and KLF family members which are involved in interleukin and inflammatory signaling (Figure 8I). Thus, we were able to show that SBNO1 binds the chromatin primarily at the TSS and may interact with TAF4 to modulate the gene expression.

## 3 Discussion

Here we demonstrated the crucial role of SBNO1 protein in cancer development and progression. We showed that SBNO1 is overexpressed in several tumor entities and utilized HCC and CCA to study the function of SBNO1. We uncovered the downstream mechanisms of SBNO1 overexpression in liver carcinogenesis by gene expression and protein‐protein interaction analysis. In particular, we showed that SBNO1 protein directly interacted with the basal transcription factor TFIID modulating RNA polymerase II activity and transcriptional regulation essential for tumor cell proliferation and clonogenicity. In HCC and iCCA mouse models, SBNO1 inhibition effectively reduced tumor growth and progression. In addition, we identified SBNO1 as an attractive therapeutic target as SBNO1 inhibition was well tolerated in mice.

Liver cancer is a heterogeneous disease primarily comprising HCC and CCA, both of which are characterized by short overall survival and limited treatment options. Combination therapy with the PD‐L1 inhibitor atezolizumab and the VEGF inhibitor bevacizumab, the combination of the anti‐PD‐L1 monoclonal antibody durvalumab and the CTLA‐4 inhibitor tremelimumab or the combination of ipilimumab (CTLA‐4 inhibitor) and nivolumab (PD‐1 inhibitor) are currently first‐line standard‐of‐care for unresectable HCC [38]. However, mean overall survival is only extended by few months and it is still unclear which patients benefit from these combination immunotherapies [38]. Only about 30% of patients with HCC respond to the combination immunotherapy and 10–15% of patients have to discontinue treatment due to treatment‐related adverse event [38]. For CCA, targeted therapeutics against FGFR2 fusion proteins (pemigatinib and futibatinib), IDH1 mutation (ivosidenib), HER2 amplification (zanidatamab) and BRAF V600E (dabrafenib/trametinib) have been approved but only a relatively small number of patients exhibit these alterations and are eligible [39]. Gemcitabine plus cisplatin and immune checkpoint inhibition is effective in a subset of patients with CCA but similar to HCC, it is unclear which patients respond [40]. Therefore, the identification of new targets for the treatment of HCC and CCA may result in improved therapeutic options.

We used four different mouse models to cover the heterogeneity of liver cancer. The first model was the previously established HCC model using intrahepatic injection of Hep55.1C into wildtype mice [41, 42]. These cells represent p53 wildtype tumors with high genomic stability. Using HDTV, HCC tumor formation was induced by Akt/Nras which represent the group of patients with Ras/MAPK pathway activation, whereas Akt/MYC tumors are dedifferentiated highly proliferative HCC and exhibit features progenitor/stem cell‐like HCC [43, 44]. Last, Akt/N1ICD‐induced iCCA show cholangiocyte‐marker expression and Notch signaling is a strong driver of human and mouse CCA [45]. Inhibition of Sbno1 significantly reduced tumor initiation and growth in all three HDTV liver cancer models demonstrating an essential role of Sbno1 in carcinogenesis.

Notch signaling has been proposed to be an attractive therapeutic target in several tumor entities [46]. Similar to p53, Ras, TGFβ and MYC, safely and effectively targeting Notch signaling has proven to be difficult suggesting that downstream effectors may be more promising [46]. To date, the function of SBNO1 and its paralog SBNO2 is still not fully uncovered. In *Drosophila*, the homolog of SBNO1 and SBNO2, sno, was identified as a downstream regulator of Notch signaling acting as a positive regulator of Notch signaling [19, 20]. In addition, sno is required for EGFR‐activated expression of the Notch ligand Delta, which induces neighboring cells to become nonneuronal cone cells, during photoreceptor cell development in *Drosophila* [47]. In mice, Sbno1 promotes cell proliferation of spermatogonial stem cells via the non‐canonical Wnt pathway [48]. During embryonal development, Sbno1 is required for Cdx2 expression and trophectoderm development [49]. Thereby, Sbno1 directly interacts with the Hippo pathway component Tead4, the FACT complex and the Notch/Rbpj transcription complex [49]. We consistently found the FACT complex component SPT16 but not SSRP1 to interact with SBNO1 in HuCCT1 cells (Table S2). Therefore, the interaction with the FACT complex may be indirect or weak. SBNO2 encodes a long and a short isoform, whereby the expression of the short isoform is induced by IL‐6/STAT3 signaling [50]. Interestingly, Brandstoetter et al. demonstrated that leukemia cells with activating STAT3 mutation have high levels of SBNO2 and depend on SBNO2 expression [51]. In concordance with these data, we showed that SBNO1 protein directly binds TAF4 of the basal transcription factor TFIID and the E‐box‐binding protein PNN. PNN‐binding relieves the transcriptional repressor CtBP and induces gene expression [52]. Our transcriptomic profiling demonstrated that SBNO1 altered transcriptional programs essential for interleukin signaling, tumor cell proliferation and clonogenicity. Additional studies are required to dissect the molecular mechanism of SBNO1/TAF4 in the regulation of target genes. These data indicate that both, SBNO1 and SBNO2, may interact with several signaling pathways altering the transcription of downstream targets. It will be interesting to test in the future if SBNO1 is an oncogenic driver.

Similar to other genes, which are promoting cancer growth, such as *Myc* and *Mdm2*, *Sbno1* knockout is embryonically lethal confirming the important function during embryogenesis [49, 53, 54]. However, SBNO1 expression is very low in most adult human tissues including the liver. We demonstrated that knockout of *Sbno1* using CRISPR/Cas9 did not induce hepatocyte cell death, liver damage nor inflammation in mice. This suggested that SBNO1 is a promising target for liver cancer therapy. Targeting SBNO1 might be feasible using small molecule inhibitors, PROTAC or other degraders effective in repressing SBNO1 protein. Over the recent years, several PROTAC have entered clinical phase II and phase III [55]. CFT8364 which targets BRD9 for degradation is evaluated in a phase III clinical trial for synovial sarcoma and the ER‐degrader ARV‐471 entered phase II for breast cancer [55]. Furthermore, PROTAC targeting MYC, STAT3 or KRAS^G12C^ have been successfully developed and entered phase I studies [55, 56]. Thus, it is feasible to target transcriptional modulators such as SBNO1 using protein degraders. Especially combination therapies may be effective by targeting tumor cell intrinsic pro‐tumorigenic pathways by SBNO1 inhibition combined with anti‐angiogenic or immunotherapy. Therefore, patients with SBNO1‐overexpressing cancers may benefit from SBNO1 inhibition.

## 4 Materials and Methods

### 4.1 Patient Samples and Clinicopathological Data

The study comprised formalin‐fixed and paraffin‐embedded (FFPE) tissue samples of 404 patients with cholangiocarcinoma (CCA) of whom 147 had intrahepatic CCA (iCCA), 140 perihilar CCA (pCCA) and 117 distal CCA (dCCA). All tissue samples were provided by the Tissue Bank of the National Center for Tumor Diseases (NCT, Heidelberg, Germany) in accordance with the regulations of the NCT Tissue Bank and informed patient consent was obtained for use of the patient material. The study protocol was approved by the ethics committee of Heidelberg University (S‐206/2005, S‐207/2005, and S‐519/2019). Each CCA tumor sample was histologically confirmed by two experienced pathologists (BG, TA). The CCA cohort consisted of adenocarcinomas only including all histologic variants and excluded combined hepatocellular cholangiocarcinoma (cHCC‐CCA). None of the patients received radio‐ and/or chemotherapy prior to surgery. Tumors were restaged according to the eighth edition of the TNM Classification of Malignant Tumors and classified according to the fifth edition of the World Health Organization (WHO) Classification of Tumors of the Digestive System [57, 58]. A summary of clinicopathological data is provided in Table S3.

### 4.2 Tissue Microarray Fabrication and Immunohistochemical Staining of SBNO1 Protein

Tissue microarray (TMA) fabrication was previously described [59]. Briefly, tissue samples from 432 patients who underwent bile duct and/or liver surgery at the University Hospital Heidelberg between 1995 and 2016 and were diagnosed with CCA were included. Tissue cores (duplicates, each 1 mm in diameter) of the marked regions were punched out from the donor blocks and embedded into a new paraffin array block using an automated tissue microarrayer (TMA Grand Master Fa. Sysmex, Germany). Due to technical issues some of the cores could not be evaluated leaving 404 patients included in this study.

### 4.3 Immunohistochemical Analysis of Human Tissue

Immunohistochemical analyses were performed on 3 µm thick TMA sections. Due to rolling, floating or detachment of tissue cores, dependent on the respective TMA and the analysis, a small number of cases could not be evaluated. Therefore, the number of samples analyzed was smaller than the total cohort. A summary of clinicopathological data of evaluated patients is included in Table S3.

For immunohistochemical staining, sections were subjected to heat‐induced epitope retrieval prior to incubation with a rabbit antibody specific for SBNO1 (Table S4). SBNO1 immunoreactivity was visualized using the Autostainer Link 48 (Dako, Agilent, Santa Clara, CA, USA) and the EnVision FLEX DAB+ Substrate Chromogen System (Dako Omnis, Agilent, Santa Clara, CA, USA). The nuclei were counterstained with hematoxylin and slides coverslipped with glycerol gelatin (both Merck, Darmstadt, Germany). SBNO1 immunoreactivity was seen in CCA tumor cells with mostly nuclear localization. For expression evaluation, a semi‐quantitative immunohistochemical assessment of SBNO1 expression was used for the normal bile duct epithelium and cancer cells, separately. Therefore, the scores of staining intensity and percentage of immunoreactive cells were calculated based on the following scoring system: the intensity ranged from 0 = negative, 1 = low, 2 = medium to 3 = high and the quantity comprised 0 = no expression, 1 = positivity in less than 10%, 2 = positivity in 10% to 50%, 3 = positivity in 51% to 80%, and 4 = positivity in more than 80% of cells. The final immunoreactive score (IRS) was obtained by multiplication of the intensity score and the quantity score ranging from 0 to 12.

### 4.4 Immunohistochemical and Histological Analysis of Murine Liver Tissue

Immunohistochemical analyses were performed on 3 µm‐thick sections of murine FFPE liver blocks. Slides were pretreated by boiling for 10 min with pH 6.0 Buffer (S2031, Agilent) or pH 9.0 Buffer (S2367, Agilent), and details of antibodies used are given in Table S4.

For anti‐Cd68, an immunoperoxidase method was used to visualize bound antibodies with DAB as chromogen (K5007, Dako REAL EnVision Detection System, Peroxidase/DAB, Rabbit/Mouse, HRP Agilent). For all other antibodies, Permanent AP Red was used (K5005, Dako REAL Detection System Peroxidase/AEC, Rabbit/Mouse, Agilent). Stained slides were scanned using the NanoZoomer slide scanner (Hamamatsu Photonics, Hamamatsu, Japan) and analyzed by QuPath digital software (https://qupath.github.io/; version 0.5.1) [60].

Whole slide images of hematoxylin and eosin (HE) stained mouse liver sections were used to determine the tumor‐to‐total liver tissue area of each slide. The total liver area analyzed was on average 2.4 cm^2^. All areas covered by tumor tissue and the total liver tissue area of each slide were measured using QuPath digital software (https://qupath.github.io/; version 0.5.1) [60].

### 4.5 Cell Lines

Eighteen human liver cancer cell lines, HuH1, HuH7, HLE, HLF, Hep3B, SNU182, SNU387, SNU475, PLC, KMCH1, HuCCT1, HuH28, RBE, SNU1079, EGI1, TFK1, SNU1196 and SNU478 and HEK293T cells were used in this study. In addition, the immortalized hepatocyte and cholangiocyte cell lines, HHT4 (provided by Curtis C. Harris) and NHC (provided by J. Banales) were analyzed. Cell lines were regularly tested for mycoplasma contamination (MycoAlert, Lonza, Basel, Switzerland), authenticated by STR analysis and cultured as described previously [61, 62]. Briefly, HuH1, HuH7, HLE, HLF, PLC, KMCH1 and HEK293T cells were cultured in Dulbecco's Modified Eagle's Medium (DMEM), Hep3B cells in Minimum Essential Medium and HuCCT1, SNU182, SNU387, HuCCT1, HuH28, RBE, SNU1079, TFK1, SNU1196 and SNU478 in RPMI1640 medium supplemented with 10% fetal bovine serum (Thermo Fisher Scientific, Offenbach, Germany) and 1% Penicillin‐streptomycin (100 IU/mL and 100 g/mL, respectively). SNU475 cells were grown in RPMI1640 medium containing 20% FBS and 1% Penicillin‐streptomycin and EGI1 in DMEM supplemented with 1% Penicillin‐streptomycin, L‐Glutamine (200 µm, Merck, Darmstadt, Germany), non‐essential amino acids (Thermo Fisher) and 10% FBS.

The two murine liver cancer cell lines Hep56.1D (Trp53 p.C132W) and Hep55.1C (Trp53 wildtype) were grown in DMEM supplemented with 1% Penicillin‐streptomycin, L‐Glutamine, and 10% FBS [23]. All media and Penicillin‐streptomycin were obtained from Sigma‐Aldrich/Merck, Darmstadt, Germany. Cell lines were transfected using Lipofectamine 2000 transfection reagent (Thermo Fisher Scientific) or polyethylenimine (PEI; Polysciences, Warrington, PA, USA) according to the manufacturer's instructions.

### 4.6 Lentiviral Transduction for SBNO1‐BirA Expression and Sbno1 CRISPR/Cas9 Knockout

To express SBNO1 protein, SBNO1 cDNA was cloned into pTRIPZ‐GW‐BirA‐C‐Flag‐Puro by Gateway cloning. For CRISPR/Cas9 knockout of murine *Sbno1*, two different sgRNA, sgSbno1.3 and sgSbno1.4, were cloned into lentiCRISPRv2. Sequences are listed in Table S5. The vector lentiCRISPRv2 was a gift from Feng Zhang (Addgene plasmid 52961; http://n2t.net/addgene:52961; RRID:Addgene_52961) [63]. All plasmid sequences were verified by Sanger sequencing.

For production of lentiviral particles, the respective lentiviral vector (10 µg) was transfected together with pMD2.G (2.5 µg; Addgene 12259) and psPAX2 (8 µg; Addgene 12260) in 1 mL Optimized Minimal Essential Medium (OptiMEM, Thermo Fisher Scientific, Waltham, MA, USA) with 60 µL Polyethylenimine (PEI; Polysciences, Warrington, PA, USA) into HEK293T cells. The medium was changed 16 h and an additional 24 h afterward, the supernatant containing lentiviral particles was filtered using a 0.45 µm Millex‐HA filter (Merck Millipore, Burlington, MA, USA) and utilized to infect the target cell lines.

### 4.7 siRNA‐Mediated Knockdown

For siRNA‐mediated knockdown, non‐targeting control (NTC) and two different siRNA targeting SBNO1 were obtained from Qiagen and sequences are listed in Table S6 (Qiagen, Germantown, MD, US). siRNA‐mediated knockdown was performed using Lipofectamine RNAiMAX Transfection Reagent (Thermo Fisher Scientific, Waltham, MA, USA) according to the manufacturer's instructions.

### 4.8 RNA Isolation and Reverse‐Transcription Polymerase Chain Reaction (qRT‐PCR)

Total RNA of cell lines was extracted with ExtractMe Total RNA Kit (Blirt, Gdansk, Poland) according to the manufacturer's protocol. cDNA was synthesized from 0.5 to 1 µg total RNA using RevertAid H Minus First Strand cDNA Synthesis Kit (Thermo Fisher Scientific). Samples of at least three independent experiments were analyzed in duplicates using primaQuant (Steinbrenner Laborsyteme GmbH, Wiesenbach, Germany) on a StepOnePlus real‐time PCR instrument (Thermo Fisher Scientific). The reference gene serine/arginine‐rich splicing factor 4 (SRSF4) was used as an internal control. Relative mRNA expression values were calculated using the comparative Ct method. Primers were obtained from Thermo Fisher Scientific and are listed in Table S7.

### 4.9 Protein Extraction and Western Blot

Proteins were extracted with cell lysis buffer (Cell Signaling Technology, Frankfurt am Main, Germany) supplemented with 1x PhosStop and 1x protease inhibitor Complete Mini EDTA‐free (Roche Diagnostics, Mannheim, Germany). Cells were lysed using sonication for 3 × 30 s with 1 min incubation on ice in between. Then, cells were centrifuged at 14 000 g at 4°C for 15 min. The protein concentration was determined using Bradford's assay according to the manufacturer's instructions (Sigma‐Aldrich, Taufkirchen, Germany). The absorbance was measured at 595 nm using the Omega FLUOStar microplate reader (BMG LABTECH, Ortenberg, Germany) and calculated based on BSA standard curve. Protein samples (25 µg) were loaded onto an 8–10% Bis/Tris polyacrylamide gel and separated by electrophoresis at 80 and 150 V for 1.5 h. Proteins were transferred onto a nitrocellulose membrane by wet blotting for 2.5 h at 90 V or overnight at 35 V and blocked with 5% milk in TBST at room temperature for 1 h. Proteins were immunoblotted with indicated antibodies (Table S8) overnight at 4°C or for 2 h at room temperature and detected with IRDye secondary antibodies using an Odyssey Sa Infrared Imaging System (LI‐COR Biosciences, Bad Homburg, Germany). Protein abundance was quantified using Image Studio v3.1.4 (LI‐COR Biosciences).

### 4.10 Cell Viability and Colony Formation

To analyze cell viability, 10 000 cells were seeded in 12‐well plates in triplicates and viability was measured every 24 h for 3–4 days. Growth medium containing 10% Resazurin (R&D Systems, Minneapolis, MN, USA) was added to the cells, incubated for 1 h at 37°C and measured (544 nm Ex/590 nm Em) with an Omega FLUOstar Microplate Reader (BMG LABTECH, Ortenberg, Germany). For colony formation, 1 000 cells were seeded in 6‐well plates in triplicates and cultured for 12–15 days. Then, cells were washed with ice‐cold PBS, fixed with 100%‐ice cold methanol for 10 min and stained with 0.5% crystal violet solution in 25% methanol for 30–60 min at room temperature. All experiments were conducted at least three times.

### 4.11 Scratch Migration Assay and Transwell Migration Assay

For scratch migration assay, Hep56.1D were infected with sgSbno1.3 or sgSbno1.4 and selected with 2 µg/mL puromycin for 3 days. Cells were seeded at 500 000 cells per well into 12‐well plates. The next day, when cells were confluent, the cell layer was scratched in a straight line using a pipette tip and cells were washed to remove detached cells. Pictures were taken at indicated time points and the area of migrating cells was quantified using FIJI software.

To perform transwell migration assays, Hep55.1C and Hep56.1D were infected with sgSbno1.3 or sgSbno1.4 and selected with 2 µg/mL puromycin. After 3 days, cells were harvested, and 75 000 cells were seeded in medium without FCS in the upper chamber of migration transwell inserts Falcon Permeable Support 8.0 µm (Corning Incorporated, NY, USA). Transwell inserts were placed in a Falcon 24‐well Companion Plate (Corning Incorporated, NY, USA) filled with 750 µL medium with 10% FCS as chemoattractant. After 24 h, the upper part of the transwell insert was swabbed gently using a cotton swab. Cells were fixed in methanol and stained with 0.5% crystal violet in 25% methanol for 1 h. The transwell inserts were thoroughly washed with double‐distilled water until the staining solution was completely removed. Pictures of the inserts were taken and the area covered by migrated cells was quantified using FIJI software. All experiments were conducted at least three times.

### 4.12 BirA‐Mediated BioID Proximity Labeling

HLF and HuCCT1 cells were infected with doxycycline (Dox)‐inducible pTRIPZ‐SBNO1‐BirA‐C‐Flag‐Puro or control pTRIPZ‐BirA‐C‐Flag‐Puro lentivirus (BirA‐Ctrl) and selected with 2 µg/mL puromycin for 3 days. For each sample, 2.5 million cells were seeded in 15 cm dishes and treated with Dox (2 µg/mL) for 24 h to induce the fusion protein. The following day, biotin (50 µm) was added to allow for the biotinylation of interacting proteins. After 24 h, cells were harvested with 1 000 µL BioID lysis buffer (500 mm Tris pH 7.5, 200 mm NaCl, 0.1% SDS, 1% Triton X‐100, 1 mM EDTA, and 0.25% sodium deoxycholate). Cells were lysed using sonication for 3 × 30 s with a 1‐min incubation on ice in between and lysates were centrifuged at 14 000 g at 4°C for 15 min.

To pull down the biotinylated proteins, 100 µL of Dynabeads MyOne Streptavidin C1 (Thermo Fisher Scientific, Waltham, MA, USA) were washed twice with 100 µL BioID lysis buffer and 1 000 µL of cell lysate was added and incubated at 4°C with rotation overnight. The beads were washed subsequently with 500 µL BioID washing buffer 1 (2% SDS), 500 µL BioID washing buffer 2 (500 mm Tris pH 7.5, 500 mm NaCl, 1% Triton X‐100, 1 mm EDTA, and 6.1% sodium deoxycholate), 500 µL BioID washing buffer 3 (10 mm Tris pH 7.5, 250 LiCl, 0.5% Triton X‐100, 1 mm EDTA, and 0.5% sodium deoxycholate), and 500 µL PBS. The bound proteins were eluted from the beads using 4x Laemmli buffer followed by incubation at 25°C for 20 min with 500 g shaking. Samples were boiled at 95°C for 8 min.

Samples were then loaded on 10% Bolt Bis‐Tris Plus gels (Thermo Fisher Scientific, Waltham, MA, USA) and run on the XCellSureLock Mini Cell Electrophoresis System (Thermo Fisher Scientific, Waltham, MA, USA) based on the provider's protocol until protein samples reached 1 cm inside the running gels. Gels were then rinsed in Millipore water and fixed using a fixation solution (50% ethanol and 10% acetic acid in water) for 30 min. After removing the fixation solution, gels were incubated in the Coomassie solution (Serva, Heidelberg, Germany) for 4 h while shaking. Gels were washed in Millipore water overnight and submitted to the Core Facility for Mass Spectrometry & Proteomics (CFMP) at the Zentrum für Molekulare Biologie der Universität Heidelberg (ZMBH) for tryptic digestion and LC/MS analysis.

### 4.13 Proximity Ligation Assay (PLA)

HuCCT1 or SNU1079 cells were seeded on 18 mm cover glasses coated with poly‐L‐lysine (PLL). Cells were fixed with 4% PFA and permeabilized with 0.2% Triton X‐100/PBS. PLA was performed according to the Naveni Proximity Ligation (Navinci, Upssala, Sweden) assay protocol. Briefly, slides were first blocked with blocking solution and incubated with two primary antibodies binding the proteins of interest overnight at 4°C. Thereby, anti‐SBNO1 raised in mouse was combined with anti‐PNN from rabbit and anti‐SBNO1 raised in rabbit was combined with anti‐TAF4, anti‐TRIM28 or anti‐PSME3 raised in mouse (Table S8). After washing with TBST, coverslips were incubated with secondary antibodies conjugated to proprietary oligonucleotide arms for 1 h at 37°C. To detect interactions, enzymatic reactions were performed subsequently at 37°C followed by washing steps in between reactions according to the instructions. Coverslips were mounted onto glass slides using the DAPI Fluoromount‐G Mounting Medium (Thermo Fisher Scientific, Waltham, MA, USA) and cells were imaged with the Nikon C2 Plus confocal microscope and Nikon Apo λS 60x NA 1.40 oil immersion objective. Image processing was conducted by FIJI software.

### 4.14 Intrahepatic Injection of Murine Cell Lines

Hep55.1C murine cells were infected with lentiviral particles encoding pFUGW‐Pol2‐Luc‐GFP and intermediate to high GFP‐positive cells were sorted to obtain homogenous luc:GFP‐fusion protein expression. pFUGW‐Pol2‐ffLuc2‐eGFP was a gift from Glenn Merlino (Addgene plasmid 71394; http://n2t.net/addgene:71394; RRID:Addgene_71394) [64].

Luc:GFP‐positive Hep55.1C cells which were derived from C57BL/6J mice [23] were injected intrahepatically into C57BL/6J mice. To induce liver tumors, 500 000 Hep55.1C cells in 25 µL 1:1‐solution of PBS and Matrigel Growth Factor Reduced (GFR) Basement Membrane Matrix (Corning/Merck) were injected into the liver parenchyma traversing several millimeters of anesthetized recipient mice after subcostal laparotomy. To monitor intrahepatic tumor growth, bioluminescent imaging (BLI) was performed once a week. Mice were injected intraperitoneally with 200 µL of 15 mg/mL Xenolight D‐luciferin (PerkinElmer, Waltham, MA, USA) and tumor growth was observed using the IVIS system.

### 4.15 Hydrodynamic Tail Vein Injection

For CRISPR/Cas9 knockout of murine *Sbno1* in vivo, the two sgRNA also used for lentiviral knockout of murine *Sbno1*, sgSbno1.3 and sgSbno1.4, were cloned into pX330‐U6‐Chimeric_BB‐CBh‐hSpCas9. pX330‐U6‐Chimeric_BB‐CBh‐hSpCas9 was a gift from Feng Zhang (Addgene plasmid 42230; http://n2t.net/addgene:42230; RRID:Addgene_42230) [65].

Hydrodynamic tail vein injection (HDTV) was performed as previously described [66]. To induce liver tumorigenesis, pT3‐EF1α‐HA‐myrAkt (Addgene plasmid 31789; http://n2t.net/addgene:31789; RRID:Addgene_31789) was injected together with pT3‐EF1α‐Nras‐G12V‐IRES‐GFP, pT3‐EF1α‐MYC (Addgene plasmid 92046; http://n2t.net/addgene:92046; RRID:Addgene_92046) or pT3‐EF1α‐N1ICD (Addgene plasmid 46047; http://n2t.net/addgene:4604; RRID:Addgene_46047). pT3‐EF1α‐NrasG12D‐IRES‐GFP was a kind gift from Darjus Tschaharganeh. Per mouse, 5 µg of each pT3 vector together with 1 µg of pCMV‐SB15 were combined with 12.5 µg pX330‐Ctrl, pX330‐sgSbno1.3 or pX330‐sgSbno1.4 in 2 mL PBS and injected into the tail veins of 8–9 weeks old FVB/N mice within 7–10 s (Charles River Laboratories, Sulzfeld, Germany). At the indicated time points, mouse livers were isolated and stored in liquid nitrogen or fixed in buffered formalin for further analysis.

The animal experiments were approved by the German Regional Council of Baden‐Wuerttemberg with approval number G‐235/18. Exclusion and termination criteria were defined by the criteria of the animal welfare officer of the University Hospital Heidelberg.

### 4.16 RNA Sequencing and Microarray

HLF and SNU1079 cells were transfected with NTC, siSBNO1#1 or siSBNO1#2 as described above. Total RNA was extracted using the Macherey‐Nagel RNA Extraction Kit (Macherey‐Nagel, Düren, Germany).

For HLF cells, gene expression profiling was performed using arrays of Clariom D Human microarrays (Thermo Fisher Scientific). Biotinylated antisense cDNA was then prepared according to the standard labelling protocol with the GeneChip WT Plus Reagent Kit and the GeneChip Hybridization, Wash and Stain Kit (both from Thermo Fisher Scientific). Afterward, the hybridization was performed in a GeneChip Hybridization oven 640, microarrays were dyed in the GeneChip Fluidics Station 450 and thereafter scanned with a GeneChip Scanner 3000. All equipment used was from Affymetrix (Affymetrix, High Wycombe, UK). Custom CDF Version 22 with ENTREZ based gene definitions was used to annotate the arrays [67]. The raw fluorescence intensity values were normalized applying quantile normalization and Kernel Surface background correction. The raw and normalized data were deposited in the GEO database (https://www.ncbi.nlm.nih.gov/geo/; accession number GSE277244).

For SNU1079 and Hep3B, 2 µg total RNA was sent to BGI Hongkong Tech Solution for mRNA sequencing using the DNBseq platform. For library preparation, oligo dT beads were used to enrich mRNA and cDNA was synthesized with respective adaptor sequences. Sequencing was performed with phi29 and single‐end 50 base reads were generated in the way of combinatorial Probe‐Anchor Synthesis. Analysis of RNA sequencing data was done with R and Bioconductor using the next generation sequencing (NGS) analysis package systempipeR [68]. The analyses of SNU1079 and Hep3B cells were conducted at different time points, and therefore, different software versions were used for data processing and analysis.

For SNU1079, quality control of raw sequencing reads was performed using FastQC (version 0.11.9). Low‐quality reads were removed using trim_galore (version 0.6.4). The resulting reads were aligned to human genome version GRCh38.p13 from GeneCode and counted using kallisto version 0.46.1 [69]. The count data was transformed to log_2_‐counts per million (logCPM), estimated the mean‐variance relationship and used this to compute appropriate observational‐level weights for linear modelling using the voom‐function from the limma package (version 3.52.2) in R (version 4.2.0) using Bioconductor (version 3.15) [70]. The raw and normalized data were deposited in the GEO database (https://www.ncbi.nlm.nih.gov/geo/; accession number GSE277271).

For Hep3B, the quality of raw sequencing reads in FASTQ format was assessed using FastQC (version 0.12.1). Low‐quality reads and adapter sequences were removed using ktrim [71]. The resulting reads were aligned to the human genome version GRCh38.p14 using the STAR aligner (version 2.7.11b) [72]. For further analysis, the counts of the chromosomal peak regions were generated using the featurecounts function from the Subread software [73]. The analysis was conducted primarily in R version 4.5.1 (2025‐06‐13) using Bioconductor 3.21 packages within the RStudio 2025.9.0.387 environment. Count values were transformed to log_2_ counts per million (log_2_‐CPM) using the cpm function from the edgeR package version 4.6.3. The raw and normalized data were deposited in the GEO database (https://www.ncbi.nlm.nih.gov/geo/; accession number GSE311232).

Differential expression analysis for all three cell lines was carried out using limma. Statistical significance was determined at a false discovery rate (FDR)–adjusted α‐level of 0.05. For pathway analyses Gene Set Enrichment Analysis (GSEA), Reactome and Qiagen Ingenuity Pathway Analysis (IPA) software were used.

### 4.17 Chromatin Immunoprecipitation (ChIP) qPCR and Sequencing

ChIP was performed using the SimpleChIP Enzymatic Chromatin IP Kit (Magnetic Beads, Cell Signaling #9003) according to the manufacturer's protocol with slight modifications as follows. For sample preparation, 4 million HuCCT1 cells were collected in 20 mL medium and subsequently crosslinked with 1% formaldehyde for 10 min. For cell lysis, samples were sonicated six times for 30 s in an ice‐cold ultrasound bath. For immunoprecipitation, samples were incubated with 1 µg of the respective antibody (Table S8) at 4°C overnight. Purified chromatin was eluted with 50 µL DNA Elution Buffer.

For qPCR, 2 µL of the purified ChIP DNA sample were used in a 10 µL reaction containing 2% DMSO and primers were added to a final concentration of 800 nM. Reactions were run in duplicate using primaQuant (Steinbrenner Laborsyteme GmbH, Wiesenbach, Germany) on a StepOnePlus real‐time PCR instrument (Thermo Fisher Scientific). The data were analyzed in Excel and GraphPad Prism 10. The information on all primers is provided in Table S7.

ChIP sequencing, 40 µL of at least 3 biological replicates for SBNO1, TAF4, and IgG control were each pooled. Each sample was precipitated using Isopropanol, resuspended in 20 µL DNase‐free water, and sent to BGI Hongkong Tech Solution for ChIP sequencing using the DNBseq platform applying single‐end sequencing with a read length of 50 bases.

The quality of raw sequencing reads in FASTQ format was assessed using FastQC (https://www.bioinformatics.babraham.ac.uk/projects/fastqc/; version 0.12.1). Low‐quality reads and adapter sequences were removed using ktrim (version 1.6.0) [71]. The resulting reads were aligned to the genome version GRCh38.p14 using bowtie2 [74]. Further analysis was conducted primarily in R version 4.5.1 (2025‐06‐13) using Bioconductor 3.21 packages within the RStudio 2025.9.0.387 environment. Peak calling was performed with normR‐function of the normr‐package (version 1.34.0). normR provides robust normalization and difference calling with a binominal mixture model [75]. The annotation of chromosomal peak regions and creating the plots was conducted with Chipseeker (version 1.44.0) [76]. Data were deposited in the GEO database (https://www.ncbi.nlm.nih.gov/geo/; accession number GSE311231).

### 4.18 Statistical Analysis

The statistical analysis of in vitro and mouse experiments was carried out using GraphPad Prism 10. Data are expressed as the mean ± SD. To compare differences between two groups, Student's t‐test or Mann–Whitney U‐test was used, as appropriate. Log‐rank test was performed to analyze patient survival. A *p*‐value < 0.05 was considered significant.

Additional Materials are available in the Supplemental Information including Figure S1 to S15 and Table S1 to S8.

## Funding

This work was supported by the German Cancer Aid (Deutsche Krebshilfe) project no. 70113922 and the German Research Foundation (DFG) project ID 314905040, 469332207 and 493697503. The authors gratefully acknowledge the data storage service SDS@hd supported by the Ministry of Science, Research and the Arts Baden‐Württemberg (MWK) and the German Research Foundation (DFG) through grant INST 35/1503‐1 FUGG.

## Conflicts of Interest

PS: grant, boards and presentations from Novartis. All other authors declare no competing interests.

## Data and Code Availability

Gene expression data of HLF, SNU1079 and Hep3B cell lines were deposited at the Gene Expression Omnibus (https://www.ncbi.nlm.nih.gov/geo/) under GSE277244, GSE277271 and GSE311232, respectively. In addition, ChIP‐seq for SBNO1 and TAF4 are available under GSE311231. All code used is available upon request.

## Supporting information




**Supporting File**: advs74197‐sup‐0001‐SuppMat.pdf.

## Data Availability

The data that support the findings of this study are openly available in GEO database at https://www.ncbi.nlm.nih.gov/geo/, reference numbers GSE277244, GSE277271, GSE311232, and GSE311231.
