# Supplementary material for: Strawberry Notch 1 Acts as a Transcriptional Regulator Driving Oncogenic Programs in Liver Carcinogenesis
Source: Adv Sci (Weinh). 2026 Feb 6;13(20):e07238. doi: 10.1002/advs.202507238 (PMC13067845; doi:10.1002/advs.202507238)
Supplement: Supplementary file 1 — Supporting File: advs74197‐sup‐0001‐SuppMat.pdf. [file ADVS-13-e07238-s001.pdf]

Supplementary Material

Strawberry Notch 1 acts as a transcriptional regulator driving oncogenic programs in liver carcinogenesis

Sarah Fritzsche *et al.*

Table of content

Supplemental Tables **2**

Table S1: Proteins significantly more abundant in the SBNO1-BirA group compared to the BirA-Ctrl in HLF cells (log_2FC>0.5, q-value≤0.05) 2

Table S2: Proteins significantly more abundant in the SBNO1-BirA group compared to the BirA-Ctrl in HuCCT1 cells (log_2FC>0.5, q-value≤0.05) 3

Table S3: Clinicopathological data of the patient cohort (N=404) 5

Table S4: Antibodies used for immunohistochemistry 6

Table S5: Sequences of sgRNA constructs targeting murine Sbno1 6

Table S6: Sequences of siRNA targeting human SBNO1 7

Table S7: Sequences of primers 7

Table S8: Antibodies used for Western blot (WB), PLA, IF and ChIP 8

Supplemental Figures **9**

Figure S1: Pan-cancer expression of *SBNO1* and *SBNO2*. 9

Figure S2: *SBNO1* and *SBNO2* mRNA and protein expression in independent CCA cohorts. 10

Figure S3: SBNO1 exhibits high expression in HCC and CCA cell lines. 11

Figure S4: Validation of gene expression profiling of HLF, Hep3B and SNU1079. 12

Figure S5: Effective reduction of Sbno1 protein by CRISPR/Cas9 in murine cells. 13

Figure S6: Sbno1 knockdown partially inhibits liver cancer initiation *in vivo*. 13

Figure S7: Tumor cell proliferation and macrophage infiltration of murine liver tumors. 14

Figure S8: Cytokeratin expression of murine liver tumors. 15

Figure S9: Expression of SBNO1 in murine and human hepatocytes. 16

Figure S10: The liver-to-body weight ratio is not altered by *Sbno1* knockout in normal liver. 17

Figure S11: BioID of SBNO1 to identify SBNO1 protein-protein interaction partners. 18

Figure S12: SBNO1 interaction partners are enriched in epigenetic transcriptional functions. 19

Figure S13: Endogenous expression of SBNO1 and its interaction partners. 20

Figure S14: Validation of SBNO1 interaction partners. 21

Figure S15: Chromatin immunoprecipitation profiles of SBNO1 and TAF4. 22

Supplemental References **23**

Supplemental Tables

Table S1: Proteins significantly more abundant in the SBNO1-BirA group compared to the BirA-Ctrl in HLF cells ($log_2FC>0.5$, q-value≤0.05)

Protein name	Accession	Log_2 FC (SBNO1 vs. Ctrl)	q-value (SBNO1 vs. Ctrl)
SBNO1	A3KN83	5.683	<0.001
ACACB	O00763	5.436	<0.001
PCNT	O95613	3.416	0.002
NKAP	Q8N5F7	3.367	0.018
TAF4	O00268	2.936	<0.001
PCCA	P05165	2.905	<0.001
SRRM2	Q9UQ35	2.892	<0.001
RPL22	P35268	2.610	0.005
CHD1L	Q86WJ1	2.548	<0.001
CKAP4	Q07065	2.485	<0.001
ZNF106	Q9H2Y7	2.426	0.007
YEATS2	Q9ULM3	2.420	<0.001
NKTR	P30414	2.412	<0.001
ASH1L	Q9NR48	2.341	0.003
ACACA	Q13085	2.132	<0.001
PRDM10	Q9NQV6	2.014	0.001
DNTTIP2	Q5QJE6	1.907	0.003
ZBTB41	Q5SVQ8	1.900	0.013
MIB1	Q86YT6	1.821	0.002
ANKRD11	Q6UB99	1.793	0.015
TAF2	Q6P1X5	1.767	0.003
SP110	Q9HB58	1.756	<0.001
LCN1	P31025	1.751	0.015
RPL26	P61254	1.732	0.025
RPL34	P49207	1.719	0.043
FABP5	Q01469	1.684	<0.001
MPHOSPH8	Q99549	1.650	0.007
POLR1A	O95602	1.575	0.011
NOP53	Q9NZM5	1.573	0.008
SON	P18583	1.527	<0.001
PHF2	O75151	1.521	0.010
DSP	P15924	1.478	0.038
PASD1	Q8IV76	1.465	0.015
DSG1	Q02413	1.358	0.037
AHDC1	Q5TGY3	1.317	0.021
NOP56	O00567	1.202	0.002
NUFIP1	Q9UHK0	1.178	0.003
BOP1	Q14137	0.953	0.013
ARG1	P05089	0.945	0.025
MCCC2	Q9HCC0	0.908	0.016
FARP1	Q9Y4F1	0.847	0.014
PNN	Q9H307	0.784	0.009
NRG1	Q02297	0.675	0.008
MCCC1	Q96RQ3	0.673	0.002
UBN1	Q9NPG3	0.650	0.040
SRRM1	Q8IYB3	0.542	0.041
ZBTB11	O95625	0.537	0.031
RRP1	P56182	0.534	0.049
TAF3	Q5VWG9	0.519	0.018

Table S2: Proteins significantly more abundant in the SBNO1-BirA group compared to the BirA-Ctrl in HuCCT1 cells ($log_2FC>0.5$, q-value≤0.05)

Protein name	Accession	Log_2 FC (SBNO1 vs. Ctrl)	q-value (SBNO1 vs. Ctrl)
SBNO1	A3KN83	7.639	<0.001
PNN	Q9H307	4.175	<0.001
TAF4	O00268	3.483	0.001
TRIM28	Q13263	3.265	0.002
SPEN	Q96T58	3.149	0.003
ZHX3	Q9H4I2	3.140	0.003
UBXN4	Q92575	3.129	0.012
PSME3	P61289	3.115	0.003
TAF6	P49848	3.103	0.003
PHC3	Q8NDX5	3.005	0.002
TAF3	Q5VWG9	2.902	0.004
MCM4	P33991	2.878	0.006
TPR	P12270	2.841	0.005
LEO1	Q8WVC0	2.838	0.005
TAF1	P21675	2.678	0.002
MASTL	Q96GX5	2.668	0.003
SRRM2	Q9UQ35	2.605	0.005
SNRNP200	O75643	2.591	0.003
RPRD2	Q5VT52	2.579	0.005
CIC	Q96RK0	2.528	0.005
SART3	Q15020	2.400	0.003
USP28	Q96RU2	2.379	0.005
GABPA	Q06546	2.360	0.006
ZNF318	Q5VUA4	2.280	0.024
XAB2	Q9HCS7	2.258	0.004
TASOR2	Q5VWN6	2.201	0.005
NCOA6	Q14686	2.179	0.017
SF3A1	Q15459	2.094	0.013
EP400	Q96L91	2.007	0.003
NCOR2	Q9Y618	2.007	0.011
SCML2	Q9UQR0	1.942	0.021
CHD1	O14646	1.907	0.012
TASOR	Q9UK61	1.855	0.043
HIVEP1	P15822	1.847	0.008
NASP	P49321	1.835	0.003
SETD2	Q9BYW2	1.819	0.018
JMJD1C	Q15652	1.813	0.004
HCFC1	P51610	1.725	0.007
RIF1	Q5UIP0	1.695	0.011
CTR9	Q6PD62	1.657	0.006
ACACA	Q13085	1.633	0.008
NCBP3	Q53F19	1.610	0.028
BRCA1	P38398	1.579	0.036
CHD8	Q9HCK8	1.569	0.011
LAMB3	Q13751	1.567	0.041
COIL	P38432	1.544	0.045
TRIM33	Q9UPN9	1.507	0.022
PUF60	Q9UHX1	1.454	0.003
SETX	Q7Z333	1.430	0.014
ZNF281	Q9Y2X9	1.427	0.010
GTF2A1	P52655	1.426	0.031
TNS4	Q8IZW8	1.422	0.045
ACIN1	Q9UKV3	1.409	0.014
BRD4	O60885	1.409	0.007
NOPCHAP1	Q8N5I9	1.407	0.046
SF3B2	Q13435	1.388	0.003

GTF2E1	P29083	1.343	0.043
TTF1	Q15361	1.333	0.015
PC	P11498	1.319	0.012
KDM3B	Q7LBC6	1.274	0.005
ZFC3H1	O60293	1.261	0.047
SAP30BP	Q9UHR5	1.180	0.043
CRNKL1	Q9BZJ0	1.087	0.026
SUGP2	Q8IX01	1.063	0.023
ZBTB4	Q9P1Z0	1.005	0.048
RIC8A	Q9NPQ8	0.946	0.008
AFF4	Q9UHB7	0.942	0.005
PRPF6	O94906	0.932	0.037
EMSY	Q7Z589	0.878	0.039
HDGFL2	Q7Z4V5	0.852	0.033
FNBP4	Q8N3X1	0.800	0.042
SUPT16H	Q9Y5B9	0.781	0.037
ZNF148	Q9UQR1	0.760	0.020
TFIP11	Q9UBB9	0.668	0.033
CPSF2	Q9P2I0	0.598	0.030
HTATSF1	O43719	0.564	0.011
IWS1	Q96ST2	0.518	0.033

Table S3: Clinicopathological data of the patient cohort (N=404).

**Parameter**	**Number (percent)**	**404 (100.0)**
**Age**	*Median years (Range)*	66 (39-81)
	Mean years (Range)	64.3(39-81)
**Sex**	*male*	264 (65.3)
	female	140 (34.7)
**UICC**[#]	*UICC 1*	14 (3.5)
	UICC 2	191 (47.3)
	UICC 3	95 (23.5)
	UICC 4	14 (3.5)
	NA	90 (22.3)
**pT**	*T1*	29 (7.2)
	T2	226 (55.9)
	T3	123 (30.4)
	T4	26 (6.4)
**pN**	*N0*	155 (38.4)
	N1	157 (38.9)
	NA	92 (22.8)
**M**	*M0*	390 (96.5)
	M1	14 (3.5)
**G**	*G1*	16 (4.0)
	G2	275 (68.1)
	G3	113 (28.0)

Table S4: Antibodies used for immunohistochemistry

Antigen	Species	Ordering no.	Company	Pretreatment	Dilution	Chromogen
SBNO1	rabbit	HPA042388	Sigma/Merck	pH6	1:200	DAB
Ki67	rabbit	ab15580	abcam	pH6	1:500	Permanent AP Red
Cd11B	rabbit	ab133357	abcam	pH6	1:1,000	Permanent AP Red
Ck7	rabbit	ab181598	abcam	pH9	1:8,000	Permanent AP Red
Ck19	rabbit	ab133496	abcam	pH9	1:100	Permanent AP Red
Hnf1b	rabbit	HPA002083	Sigma/Merck	pH6	1:200	Permanent AP Red
Hnf4a	rabbit	MA5-14891	Thermo Fisher Scientific	pH6	1:100	Permanent AP Red
Cd105	goat	AF1320-SP	Bio-techne	pH6	1:1,000	Permanent AP Red
pH2A.X	rabbit	9718S	Cell Signaling	pH6	1:100	Permanent AP Red
Hmgb1	rabbit	ab79823	abcam	pH6	1:200	Permanent AP Red
Cd68	rat	137001	Biolegend	pH6	1:100	DAB

Table S5: Sequences of sgRNA constructs targeting murine Sbno1

Gene	sgRNA	siRNA target sequence 5'-3'	Supplier
Sbno1	sgSbno1.3 fw	CACC G CAGCACGGTCTCCATTAGGT	Thermo Fisher Scientific
Sbno1	sgSbno1.3 rev	AAAC ACCTAATGGAGACCGTGCTG C	Thermo Fisher Scientific
Sbno1	sgSbno1.4 fw	CACC GTATAACTCTACATGGCCGG	Thermo Fisher Scientific
Sbno1	sgSbno1.4 rev	AAAC CCGGCCATGTAGAGTTATAC	Thermo Fisher Scientific

Table S6: Sequences of siRNA targeting human SBNO1

Gene	siRNA	siRNA target sequence 5'-3'	Supplier
SBNO1	siSBNO1#1	TCGGACGTACTCATAGATCAA	Qiagen, SI00711256
SBNO1	siSBNO1#2	GACGATAGCAGGAATCATCTA	Qiagen, SI04164258

Table S7: Sequences of primers

Gene	Application	Sequence 5'-3'	Amplicon
KIT	qRT-PCR	Fwd: CGTTCTGCTCCTACTGCTTCG<br>Rev: CCCACGCGGACTATTAAGTCT	117 bp
LIF	qRT-PCR	Fwd: CCAACGTGACGGACTTCCC<br>Rev: TACACGACTATGCGGTACAGC	82 bp
LIF	ChIP-qPCR	Fwd: TATGGGCTGCACTTCAGAGGGC<br>Rev: GGACTGGGGATCCCGGCTAA	150 bp
MCM2	qRT-PCR	Fwd: CATCAGCGACATGTGCAAAG<br>Rev: GTTCACCACCAGGCTCTCAC	46 bp
MCM2	ChIP-qPCR	Fwd: GGCTCTTCCCGGGCTTTGGT<br>Rev: ACCACGATCCTCTCCGCCAC	85 bp
RELN	qRT-PCR	Fwd: ACTGTGCACCCACATCTAGC<br>Rev: TAATCGCGCCACACTGTTCT	145 bp
SBNO1	qRT-PCR	Fwd: TCGCCTCTGGTCCTTCAAAC<br>Rev: AACTGACGGGGTAGGCATTG	194 bp
SRSF4	qRT-PCR	Fwd: TGCAGCTGGCAAGACCTAAA<br>Rev: TTTTTGCGTCCCTTGTGAGC	80 bp
TCF21	qRT-PCR	Fwd: TCCTGGCTAACGACAAATACGA<br>Rev: TTTCCCGGCCACCATAAAGG	77 bp

Table S8: Antibodies used for Western blot (WB), PLA, IF and ChIP

Antigen	Species	Ordering no.	Company	Application	Dilution
β-actin	mouse	691001	Mpbio	WB	1:10,000
β-actin	rabbit	4970S	Cell Signaling	WB	1:10,000
Biotin	mouse	M743	Dako	WB	1:1,000
PNN/Pinin	rabbit	18266-1-AP	Proteintech	IF, PLA	1:50
PSME3/PA28γ	mouse	sc-136025	Santa Cruz	IF, PLA	1:50
				WB	1:1,000
SBNO1	rabbit	HPA042388	Sigma/Merck	IF, PLA	1:50
				ChIP	1 µg
SBNO1	mouse	sc-166519	Santa Cruz	IF, PLA	1:50
TAF4	mouse	MA3-075	Thermo Fisher Scientific	IF, PLA	1:100
TAF4	rabbit	PA540711	Thermo Fisher Scientific	ChIP	1 µg
IgG	rabbit	2729	Cell Signaling	ChIP	1 µg
TRIM28/TIF1b (D-7)	mouse	sc-515790	Santa Cruz	IF, PLA	1:100
IRDye 680LT anti-mouse IgG	donkey	926-68022	LI-COR Biosciences	WB	1:10,000
IRDye 680LT anti-rabbit IgG	donkey	926-68023	LI-COR Biosciences	WB	1:10,000
IRDye 800CW anti-mouse IgG	donkey	926-32212	LI-COR Biosciences	WB	1:10,000
IRDye 800CW anti-rabbit IgG	donkey	926-32213	LI-COR Biosciences	WB	1:10,000
Cy™3 AffiniPure Anti-Mouse IgG	donkey	715-165-150	Jackson ImmunoResearch	IF	1:500
Alexa Fluor® 488 AffiniPure Anti-Mouse IgG	donkey	715-545-150	Jackson ImmunoResearch	IF	1:250

Supplemental Figures

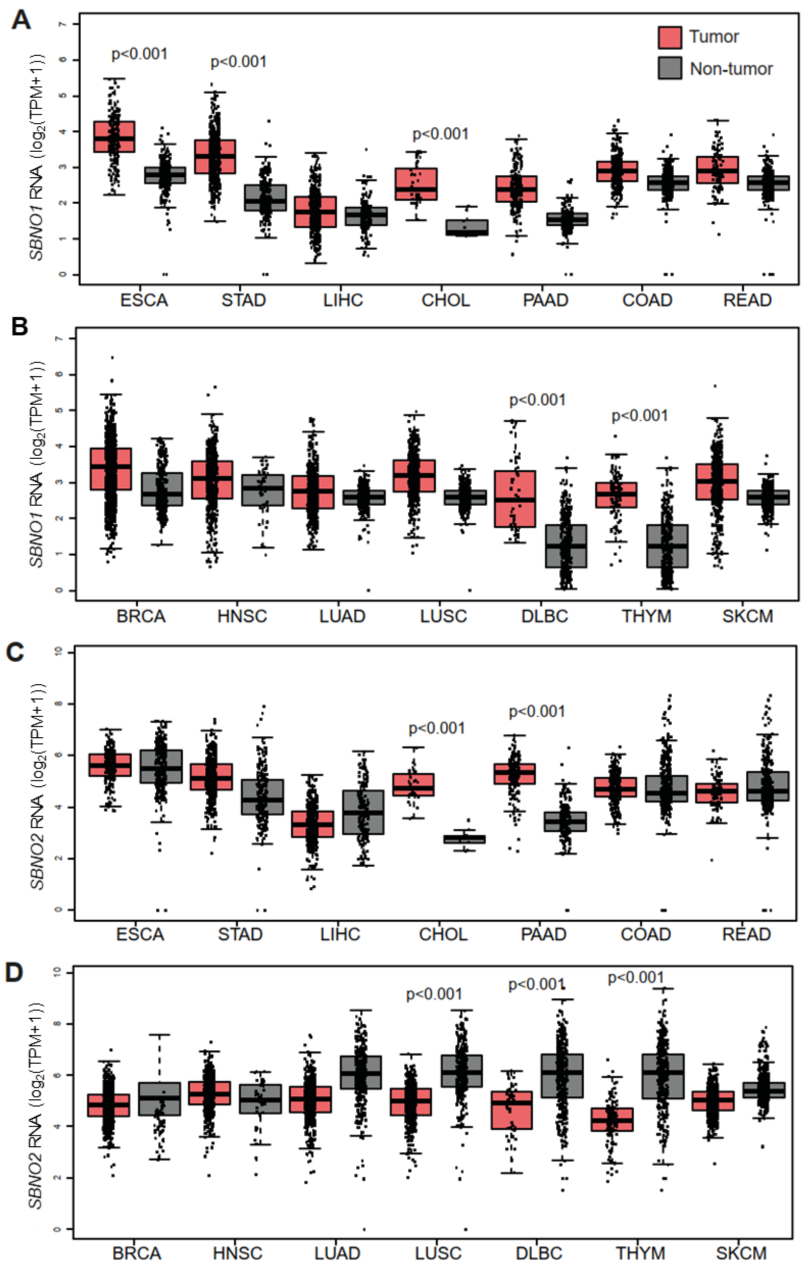

Figure S1: Pan-cancer expression of *SBNO1* and *SBNO2*.

Expression of *SBNO1* in tumor and non-tumor samples of TCGA and GTEx obtained from GEPIA (http://gepia.cancer-pku.cn/). **(A)** *SBNO1* mRNA levels in esophageal carcinoma (ESCA), stomach adenocarcinoma (STAD), hepatocellular carcinoma (LIHC), cholangiocarcinoma (CHOL), pancreatic adenocarcinoma (PAAD), colon adenocarcinoma (COAD) and rectum adenocarcinoma (READ). Tumor is indicated in red and non-tumor in grey. **(B)** *SBNO1* mRNA levels in breast cancer (BRCA), head and neck squamous cell carcinoma (HNSC), lung adenocarcinoma (LUAD), lung squamous cell carcinoma (LUSC), diffuse large B-cell lymphoma (DLBC), thymoma (THYM) and melanoma (SKCM). **(C)** *SBNO2* mRNA levels in ESCA, STAD, LIHC, CHOL, PAAD, COAD and READ. **(D)** *SBNO2* mRNA levels in BRCA, HNSC, LUAD, LUSC, DLBC, THYM and SKCM. *P*-values were calculated using unpaired Student's t-test.

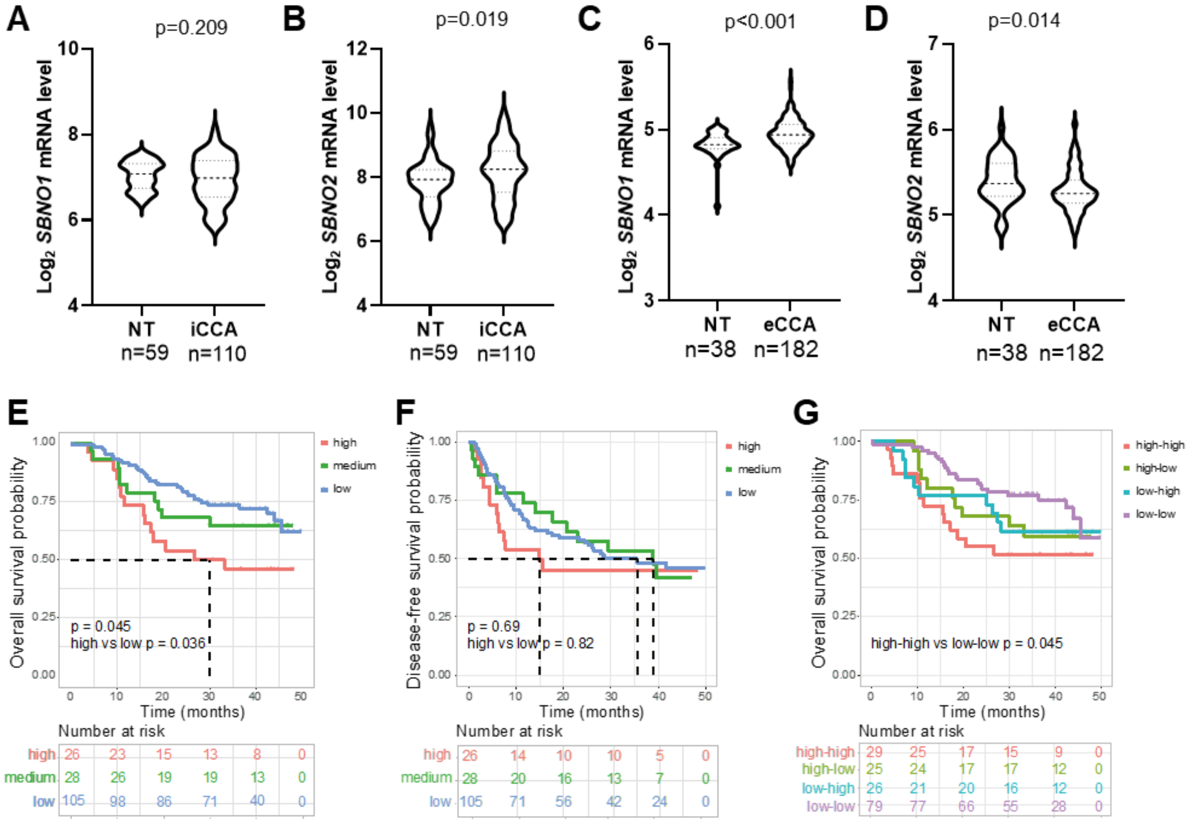

Figure S2: *SBNO1* and *SBNO2* mRNA and protein expression in independent CCA cohorts.

(A) *SBNO1* and **(B)** *SBNO2* mRNA levels in non-tumor and tumor tissue samples of patients with iCCA (GSE26566) [1]. **(C)** The expression of *SBNO1* and **(D)** *SBNO2* mRNA of patients with eCCA is depicted (GSE132305) [2]. *P*-values were calculated using unpaired Student's t-test. **(E)** Overall survival and **(F)** disease-free survival of patients with iCCA exhibiting low, medium or high SBNO1 protein levels (PDC000198). **(G)** Overall survival of patients with high or low SBNO1 protein and mRNA levels. *P*-values for survival analysis were calculated using log-rank test.

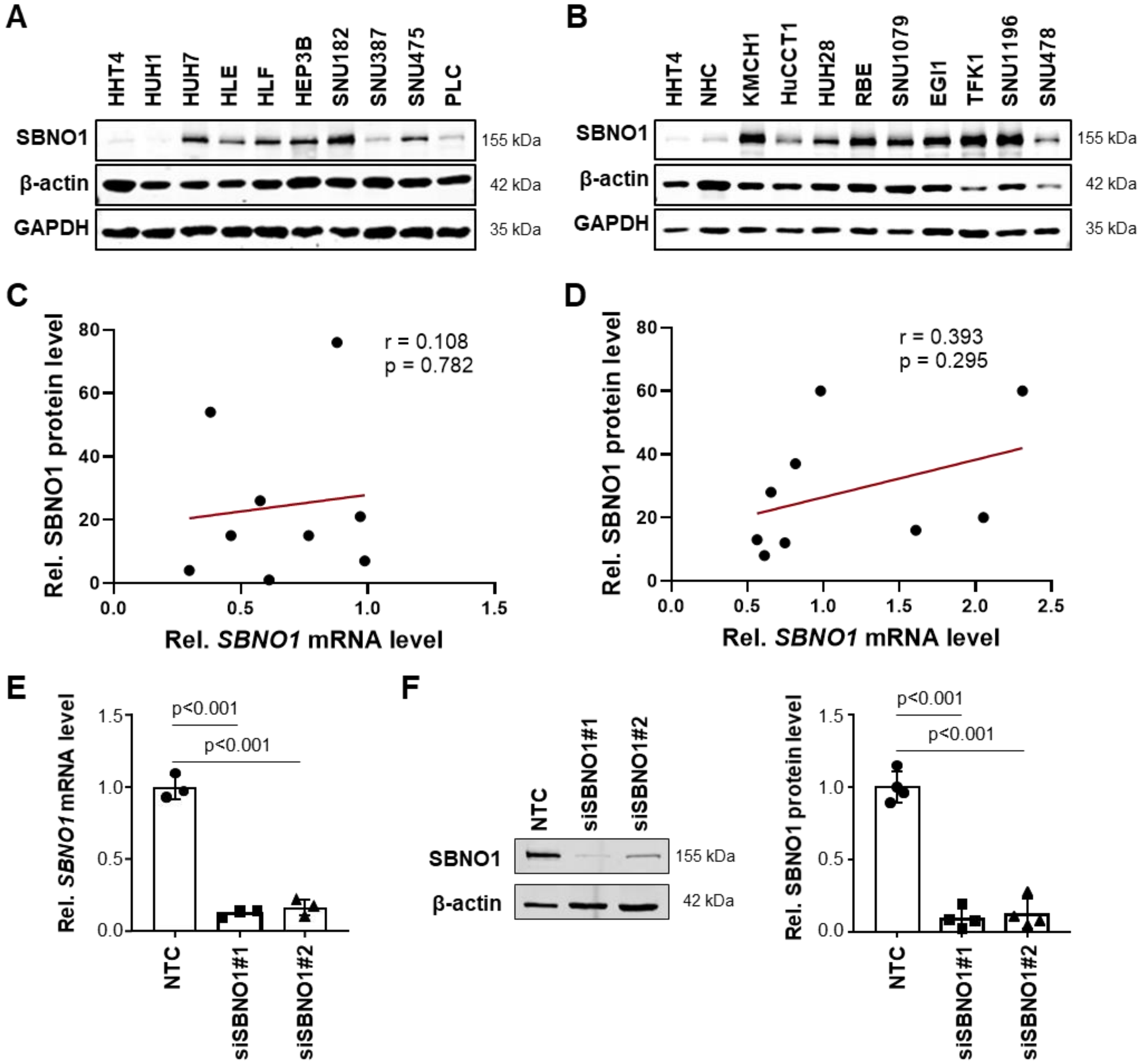

Figure S3: SBNO1 exhibits high expression in HCC and CCA cell lines.

Expression of SBNO1 protein in normal hepatocyte (HHT4), normal cholangiocyte (NHC), HCC **(A)** and CCA **(B)** cell lines. GAPDH and β-actin served as loading controls. **(C)** Correlation analysis of SBNO1 protein and mRNA levels in HCC (n=9) and **(D)** CCA (n=9) cell lines. **(E)** Efficient knockdown of *SBNO1* mRNA analyzed by qRT-PCR using the two siRNAs siSBNO1#1 and siSBNO1#2 and the non-targeting control (NTC) in the HCC cell line HLF (n=3). **(F)** Validation of reduced SBNO1 protein levels upon siRNA transduction of HLF cells by Western blot. Shown is one representative Western blot. Quantification of SBNO1 Western blots normalized to β-actin relative to the non-targeting control (NTC; n=4). *P*-values were calculated using unpaired Student's t-test.

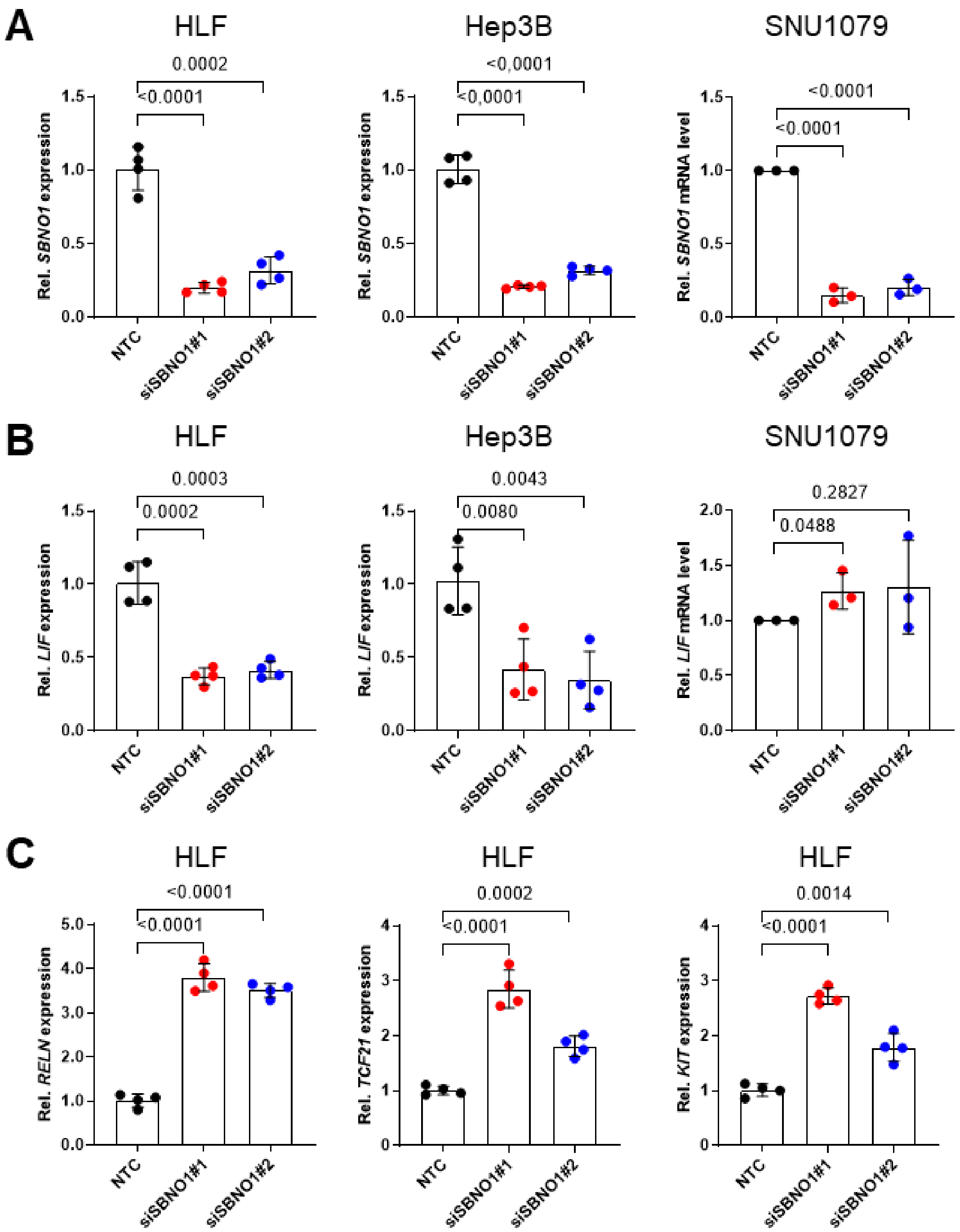

Figure S4: Validation of gene expression profiling of HLF, Hep3B and SNU1079.

(A) qRT-PCR analysis of *SBNO1* and **(B)** *LIF* mRNA expression in HLF (n=4), Hep3B (n=4) and SNU1079 (n=3) cells upon transfection with siRNA control (NTC), siSBNO1#1 or siSBNO1#2, as indicated. **(C)** qRT-PCR analysis of *RELN*, *TCF21* and *KIT* mRNA expression upon siRNA transduction with siRNA control (NTC), siSBNO1#1 or siSBNO1#2 of HLF cells (n=4). *P*-values were calculated using unpaired Student's t-test.

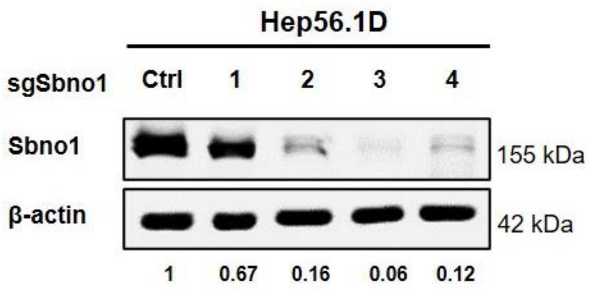

Figure S5: Effective reduction of Sbno1 protein by CRISPR/Cas9 in murine cells.

Western blot of Sbno1 protein confirming efficient Sbno1 protein knockout by CRISPR/Cas9 using guide RNA sgSbno1.2, sgSbno1.3 or sgSbno1.4 in the murine cell line Hep56.1D. β-actin served as loading control. Numbers indicate the Sbno1 protein levels normalized to β-actin relative to control cells (Ctrl).

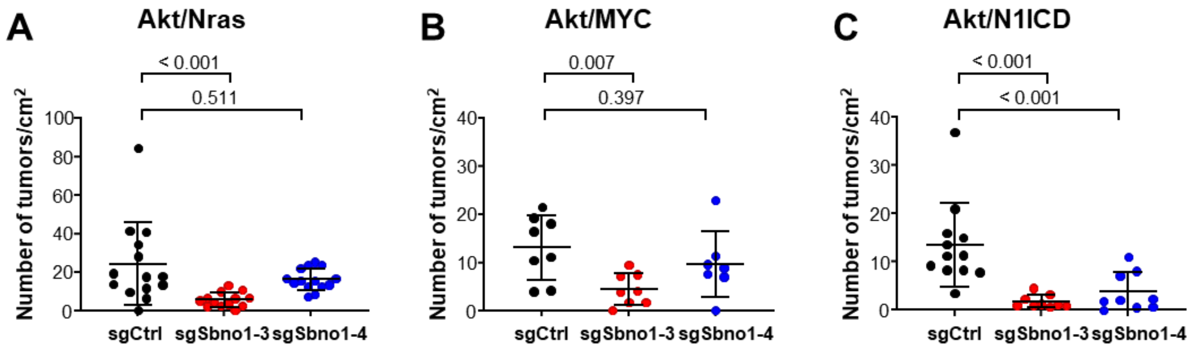

Figure S6: Sbno1 knockdown partially inhibits liver cancer initiation *in vivo*.

The number of tumor nodules was counted on whole slide images for three mouse models using HDTV. **(A)** Mice were transduced with myrAkt/Nras-G12V (Akt/Nras) together with sgCtrl (n=14), sgSbno1.3 (n=13) or sgSbno1.4 (n=14). **(B)** Akt/MYC together with sgCtrl (n=8), sgSbno1.3 (n=8) or sgSbno1.4 (n=7) was injected. **(C)** Shown are livers transduced with Akt/N1ICD together with sgCtrl (n=12), sgSbno1.3 (n=9) or sgSbno1.4 (n=9). **(A-C)** Livers were collected 9-11 weeks, 5 weeks or 8 weeks after injection for the Akt/Nras, Akt/MYC and Akt/N1ICD mouse model, respectively. Mann–Whitney U-test *P*-values are depicted.

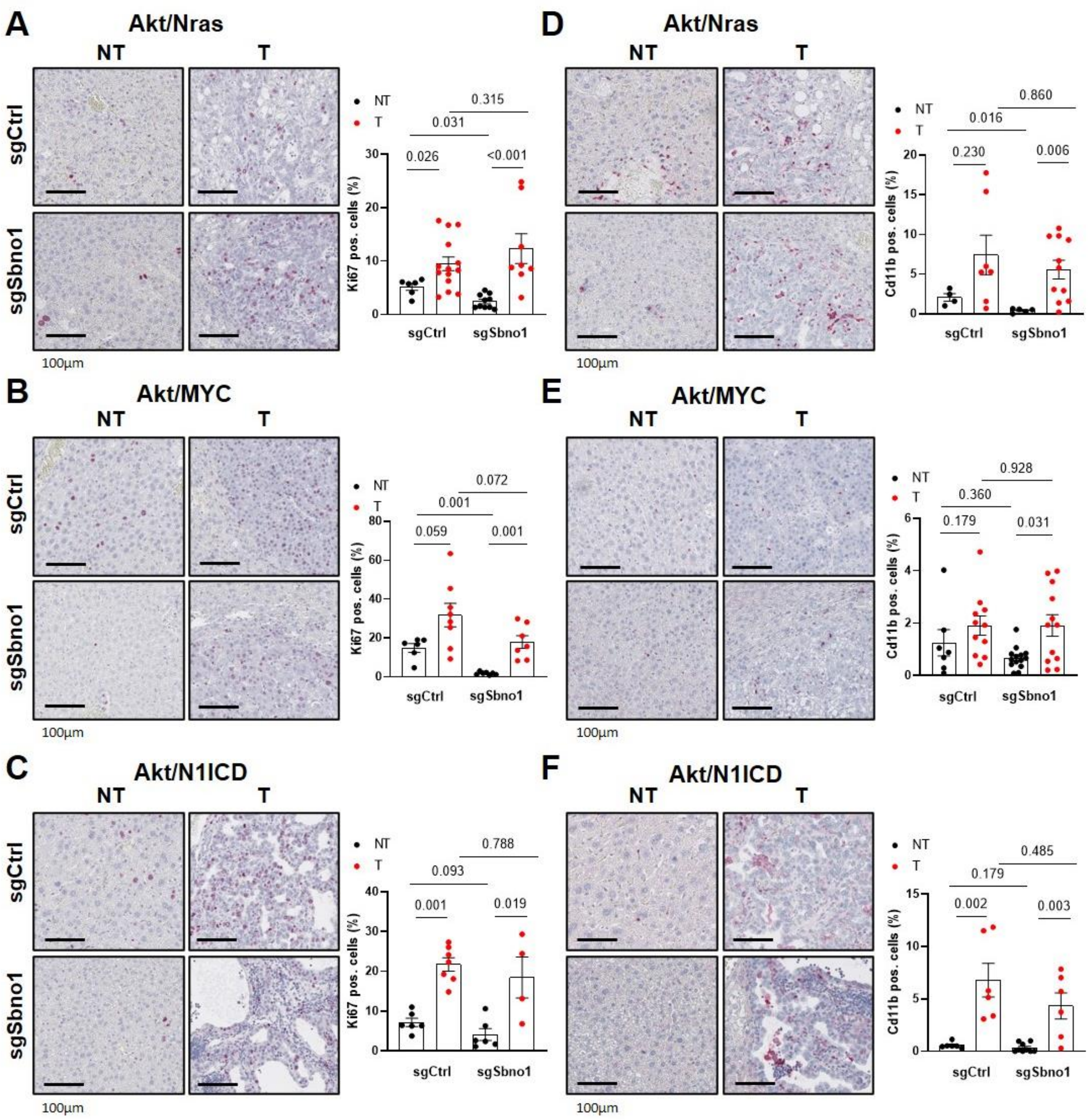

Figure S7: Tumor cell proliferation and macrophage infiltration of murine liver tumors.

(A) Representative anti-Ki67 immunohistochemical staining of murine liver sections of mice injected with Akt/Nras, **(B)** Akt/MYC and **(C)** Akt/N1ICD together with sgCtrl or sgSbno1 as indicated. **(D)** Representative anti-Cd11b immunohistochemical staining of murine liver sections of mice injected with Akt/Nras, **(E)** Akt/MYC and **(F)** Akt/N1ICD together with sgCtrl or sgSbno1 as indicated. Data of both sgSbno1 (SgSbno1#3 and sgSbno1#4) were combined as they showed similar results. NL, normal liver; T, tumor. Mann–Whitney U-test *P*-values are depicted.

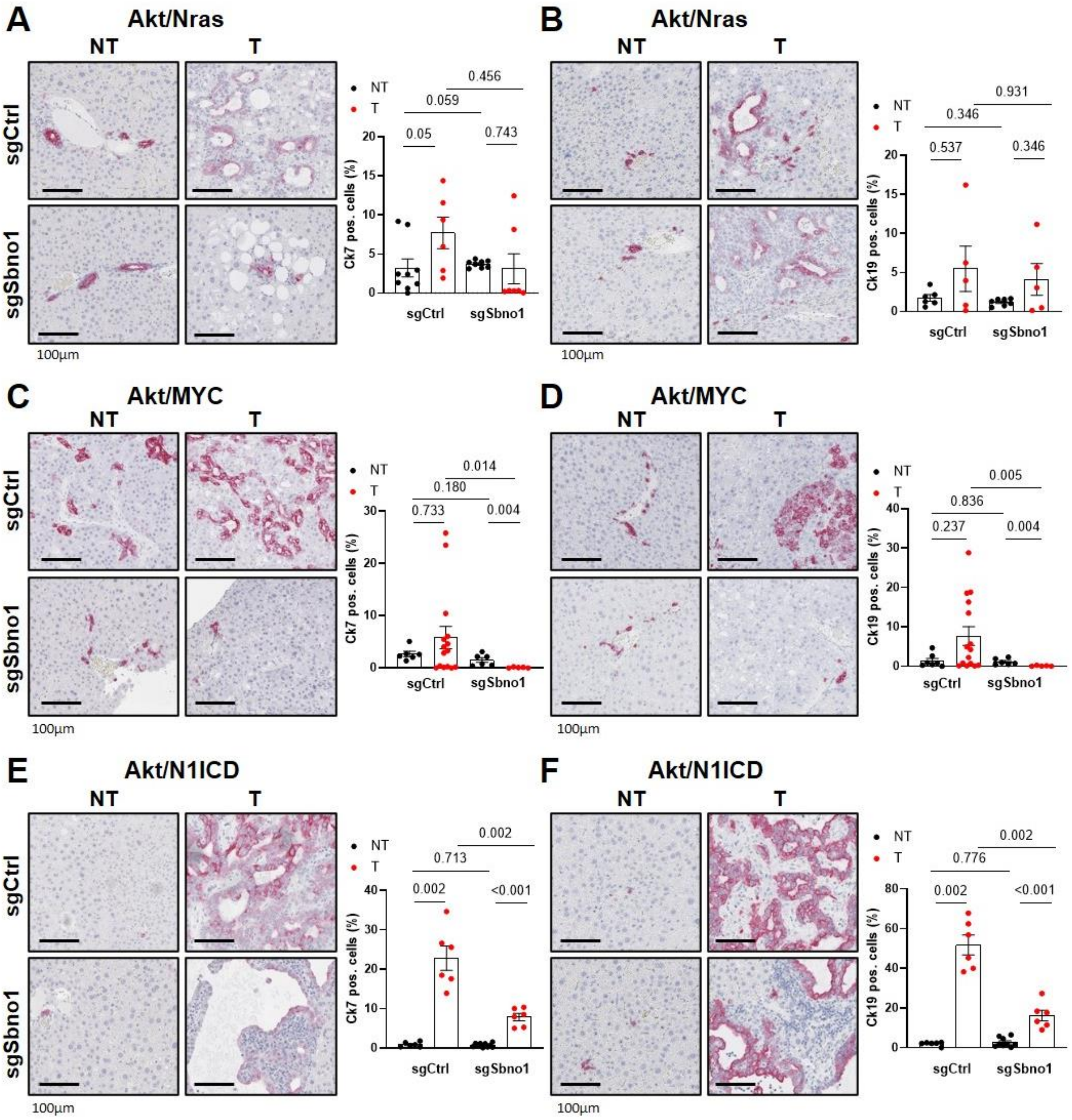

Figure S8: Cytokeratin expression of murine liver tumors.

(A) Representative anti-Ck7 and **(B)** anti-Ck19 immunohistochemical staining of murine liver sections of mice injected with Akt/Nras. **(C)** Expression of Ck7 and **(D)** Ck19 in the Akt/MYC and **(E** and **F)** the Akt/N1ICD mouse model. The percentage of positive cells in non-tumor (NT) and tumor (T) areas of mice in the control group (sgCtrl) or sgSbno1 group are as indicated. Data of both sgSbno1 (SgSbno1.3 and sgSbno1.4) were combined as they showed similar results. NT, normal liver; T, tumor. Mann–Whitney U-test *P*-values are depicted.

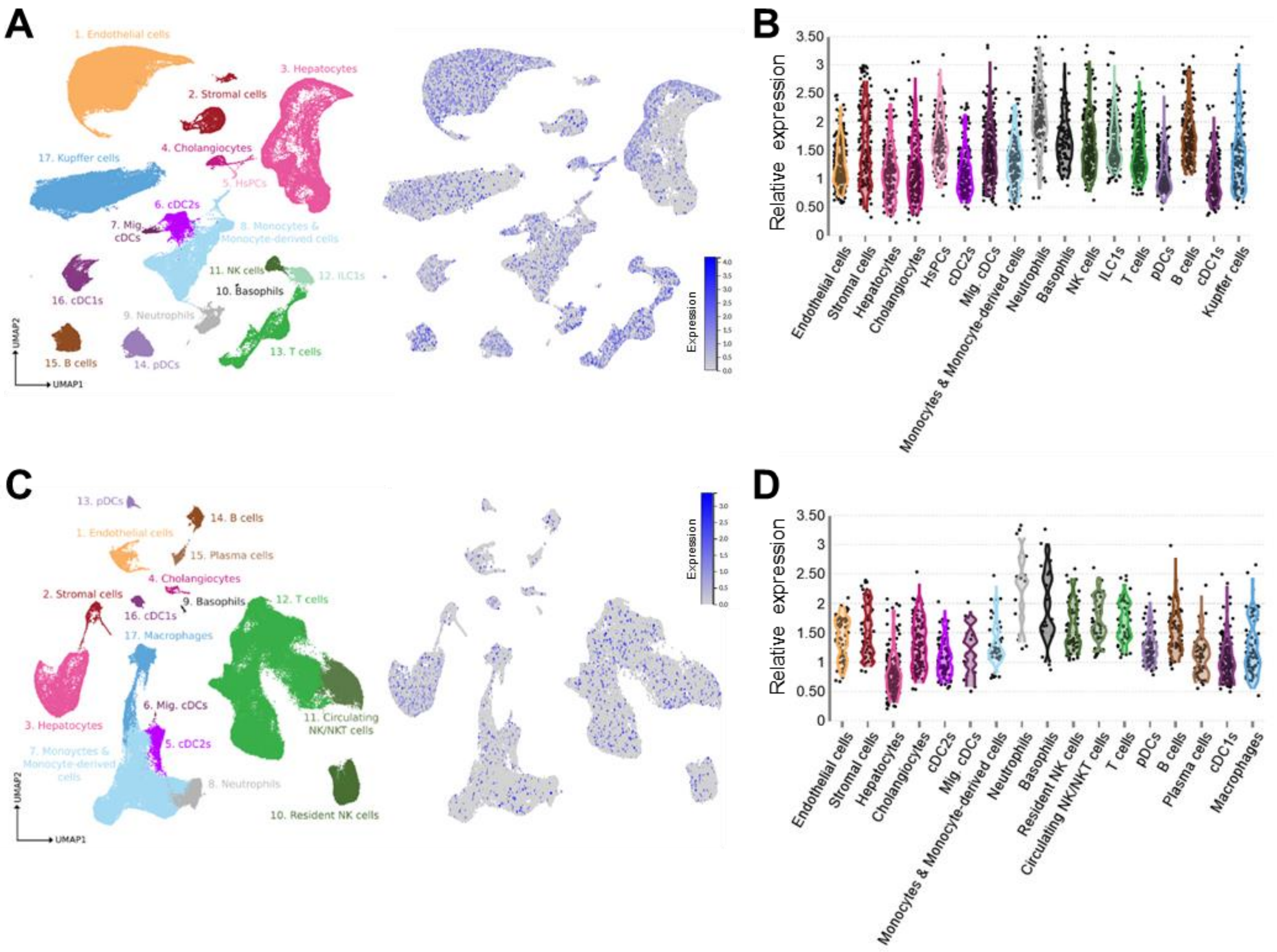

Figure S9: Expression of SBNO1 in murine and human hepatocytes.

(A) UMAP plot of murine liver cells using sc/snRNA-seq data (https://www.livercellatlas.org; GSE192742) showing the cell type annotation (left panel) and the expression of Sbno1 in the cells of the murine liver (right panel) [3]. **(B)** Violin plots depicting the expression of Sbno1 in the murine liver cell types. **(C)** UMAP plot of human liver cells using sc/snRNA-seq data (https://www.livercellatlas.org) showing the cell type annotation (left panel) and the expression of SBNO1 in the cells of the human liver (right panel). **(D)** Violin plots depicting the expression of SBNO1 in the human liver cell types.

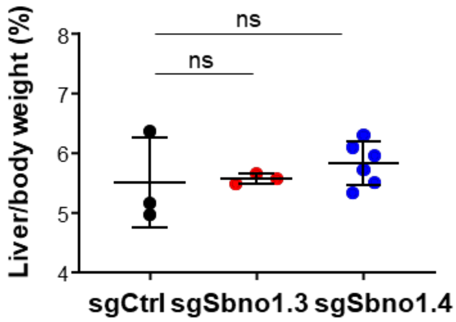

Figure S10: The liver-to-body weight ratio is not altered by *Sbno1* knockout in normal liver.

The liver-to-body weight ratio of mice one week after *Sbno1* knockout by sgSbno1.3 or sgSbno1.4 compared to control sgCtrl without induction of liver cancer is shown.

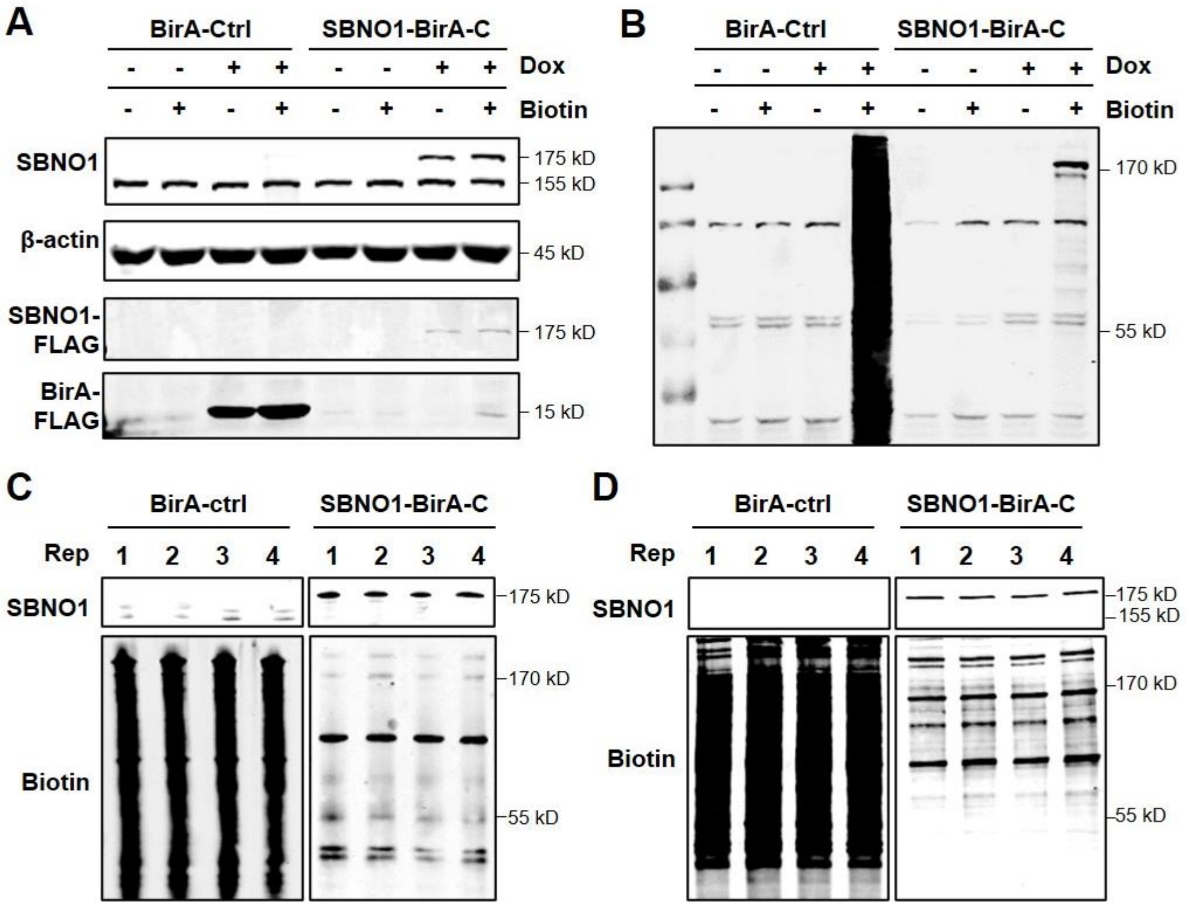

Figure S11: BioID of SBNO1 to identify SBNO1 protein-protein interaction partners.

(A) HLF cells were infected with SBNO1-BirA fusion or BirA-Ctrl lentiviral construct. Protein expression was induced by incubation with Dox (2 µg/mL) and interaction partners were labelled with biotin by BirA upon incubation of cells with biotin (50 µM) for 24 h. Western blots showing SBNO1, SBNO1-FLAG and BirA-FLAG as indicated. β-actin served as loading control. **(B)** In addition, biotinylated proteins were detected by anti-Biotin Western blot. **(C)** Biotinylated proteins were detected with anti-Biotin antibody in four biological replicates of the input samples (left panel) and after streptavidin pulldown (right panel) of HLF cells and **(D)** of HuCCT1 cells. Four biological replicates were prepared for each construct and subjected to mass-spectrometric analysis.

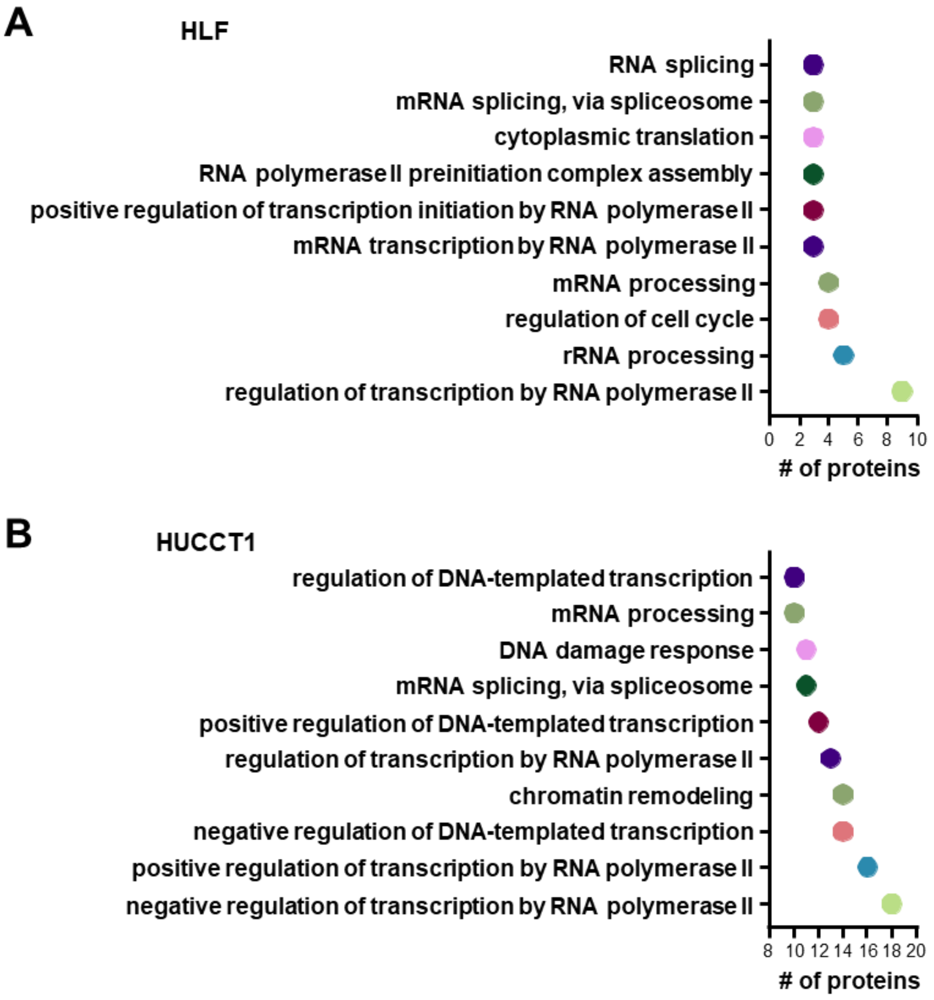

Figure S12: SBNO1 interaction partners are enriched in epigenetic transcriptional functions.

(A) Significantly biotinylated proteins with $log_2FC>0.5$ were analyzed using the DAVID tool (https://davidbioinformatics.nih.gov/) for enrichment in biological processes in HLF and **(B)** HuCCT1 cells. Shown are the top 10 enriched biological processes of each cell lines.

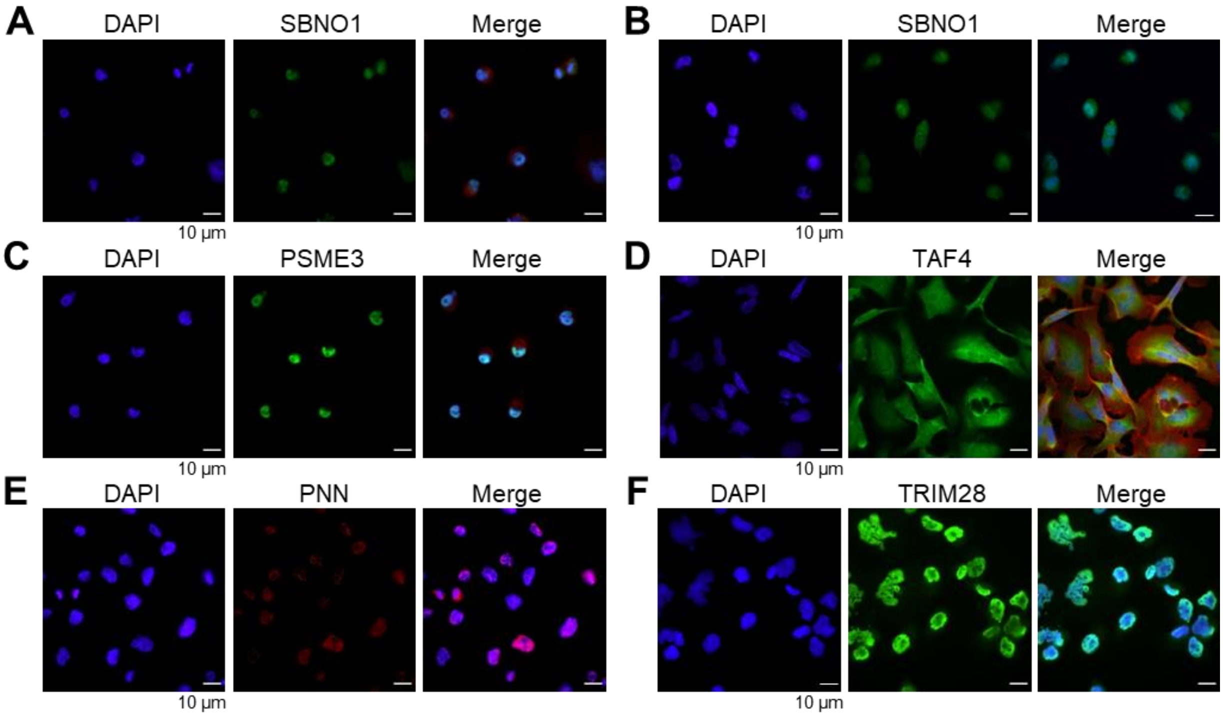

Figure S13: Endogenous expression of SBNO1 and its interaction partners.

(A) Representative images of immunofluorescence detecting SBNO1 protein using a rabbit or **(B)** mouse anti-SBNO1 antibody. **(C)** Expression of PSME3, **(D)** TAF4, **(E)** PNN and **(F)** TRIM28 by immunofluorescence. DAPI was used for nuclear staining. All images were obtained in HuCCT1 cells. rb: anti-rabbit antibody, ms: anti-mouse antibody.

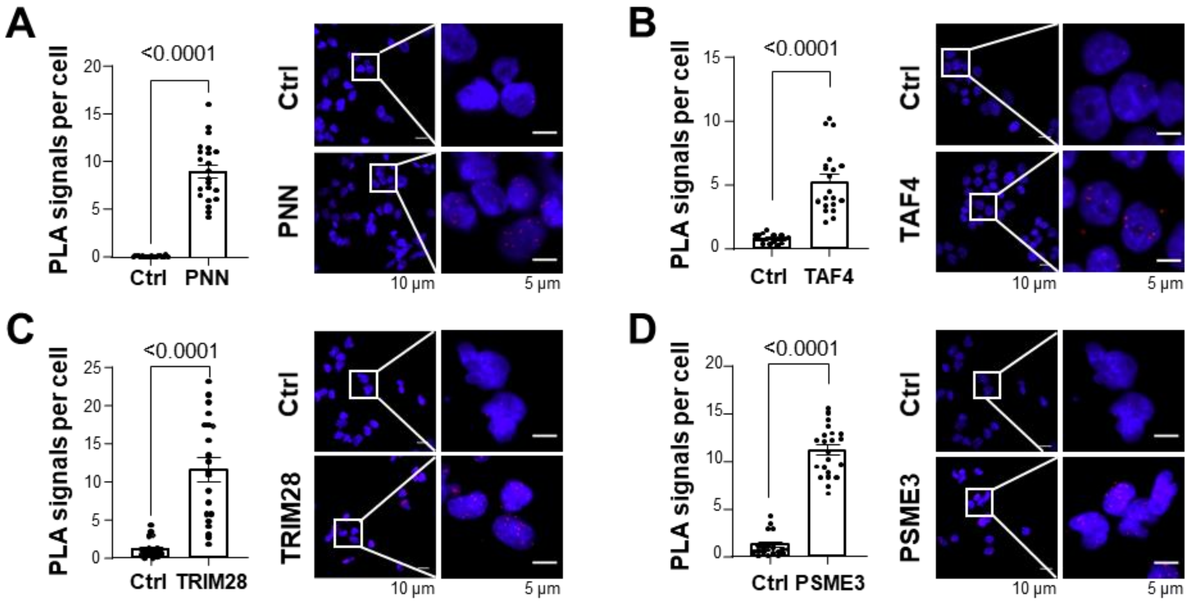

Figure S14: Validation of SBNO1 interaction partners.

(A) Quantification and representative images of PLA for interaction of SBNO1 with PNN, **(B)** TAF4, **(C)** TRIM28 and **(D)** PSME in SNU1079 cells. Quantitative representation of PLA dots per cell by scatter plots with mean and SD. N = 20 cells of 8 images analyzed for each condition are depicted. Mann–Whitney U-test *P*-values are shown.

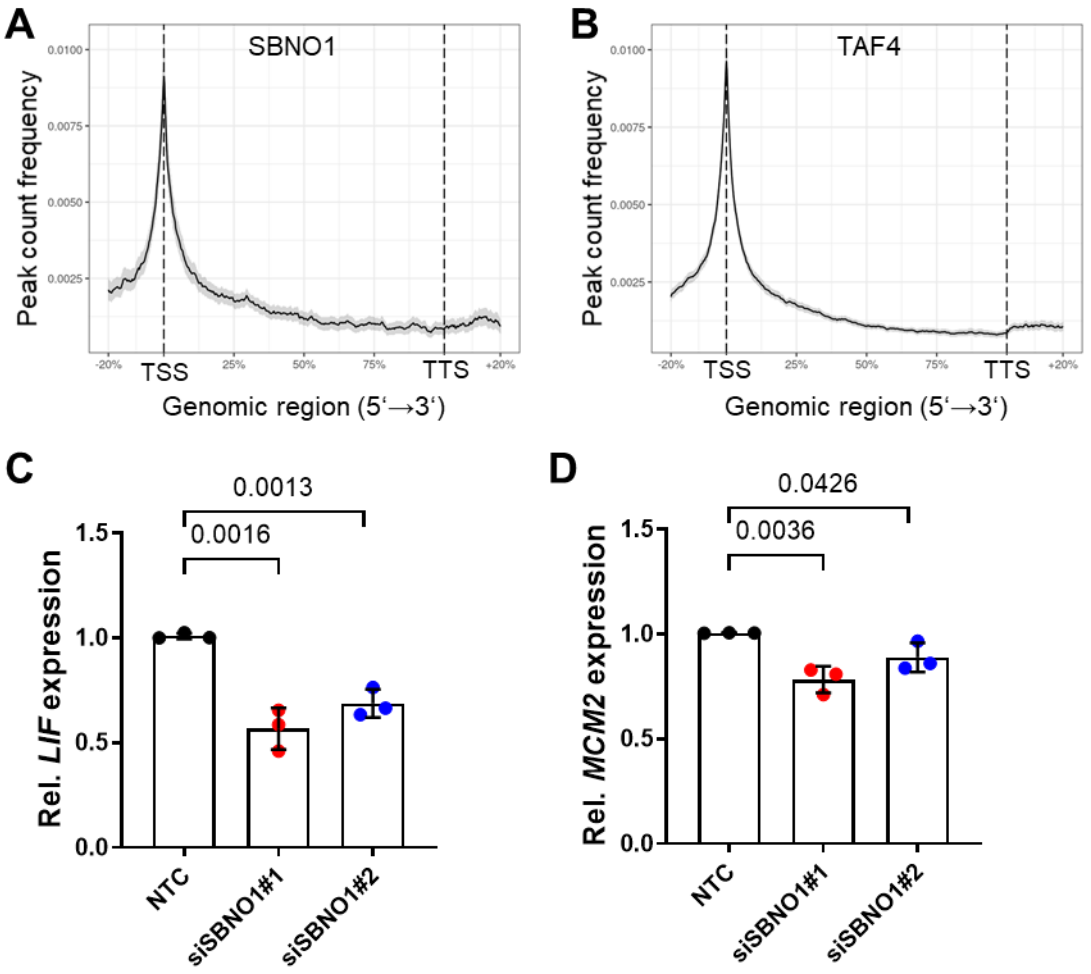

Figure S15: Chromatin immunoprecipitation profiles of SBNO1 and TAF4.

(A) Chromatin immunoprecipitation sequencing (ChIP-seq) enrichment analysis along the genomic region of genes showed an enrichment peak at the transcription start site (TSS) for SBNO1 and **(B)** TAF4 ChIP-seq, respectively. TTS: transcription termination site. **(C)** qRT-PCR analysis of *LIF* and **(D)** *MCM2* mRNA expression in HuCCT1 cells (n=3) upon transfection with siRNA control (NTC), siSBNO1#1 or siSBNO1#2. *P*-values were calculated using unpaired Student's t-test.

Supplemental References

[1] J. B. Andersen, B. Spee, B. R. Blechacz, I. Avital, M. Komuta, A. Barbour, E. A. Conner, M. C. Gillen, T. Roskams, L. R. Roberts, V. M. Factor, S. S. Thorgeirsson, Genomic and genetic characterization of cholangiocarcinoma identifies therapeutic targets for tyrosine kinase inhibitors. *Gastroenterology* **2012**, *142* (4), 1021, https://doi.org/10.1053/j.gastro.2011.12.005.

[2] R. Montal, D. Sia, C. Montironi, W. Q. Leow, R. Esteban-Fabro, R. Pinyol, M. Torres-Martin, L. Bassaganyas, A. Moeini, J. Peix, L. Cabellos, M. Maeda, C. Villacorta-Martin, P. Tabrizian, L. Rodriguez-Carunchio, G. Castellano, C. Sempoux, B. Minguez, T. M. Pawlik, I. Labgaa, L. R. Roberts, M. Sole, M. I. Fiel, S. Thung, J. Fuster, S. Roayaie, A. Villanueva, M. Schwartz, J. M. Llovet, Molecular classification and therapeutic targets in extrahepatic cholangiocarcinoma. *J Hepatol* **2020**, *73* (2), 315, https://doi.org/10.1016/j.jhep.2020.03.008.

[3] M. Guilliams, J. Bonnardel, B. Haest, B. Vanderborght, C. Wagner, A. Remmerie, A. Bujko, L. Martens, T. Thone, R. Browaeys, F. F. De Ponti, B. Vanneste, C. Zwicker, F. R. Svedberg, T. Vanhalewyn, A. Goncalves, S. Lippens, B. Devriendt, E. Cox, G. Ferrero, V. Wittamer, A. Willaert, S. J. F. Kaptein, J. Neyts, K. Dallmeier, P. Geldhof, S. Casaert, B. Deplancke, P. Ten Dijke, A. Hoorens, A. Vanlander, F. Berrevoet, Y. Van Nieuwenhove, Y. Saeys, W. Saelens, H. Van Vlierberghe, L. Devisscher, C. L. Scott, Spatial proteogenomics reveals distinct and evolutionarily conserved hepatic macrophage niches. *Cell* **2022**, *185* (2), 379, https://doi.org/10.1016/j.cell.2021.12.018.
